# Supplementary material for: HIV-1 Envelope and MPER Antibody Structures in Lipid Assemblies
Source: Cell Rep. 2020 Apr 28;31(4):107583. doi: 10.1016/j.celrep.2020.107583 (PMC7196886; doi:10.1016/j.celrep.2020.107583)
Supplement: Document S1. Figures S1–S6 and Table S1 [file mmc1.pdf]

Cell Reports, Volume *31*

Supplemental Information

HIV-1 Envelope and MPER Antibody

Structures in Lipid Assemblies

Kimmo Rantalainen, Zachary T. Berndsen, Aleksandar Antanasijevic, Torben Schiffner, Xi Zhang, Wen-Hsin Lee, Jonathan L. Torres, Lei Zhang, Adriana Irimia, Jeffrey Copps, Kenneth H. Zhou, Young D. Kwon, William H. Law, Chaim A. Schramm, Raffaello Verardi, Shelly J. Krebs, Peter D. Kwong, Nicole A. Doria-Rose, Ian A. Wilson, Michael B. Zwick, John R. Yates III, William R. Schief, and Andrew B. Ward

Supplemental Information

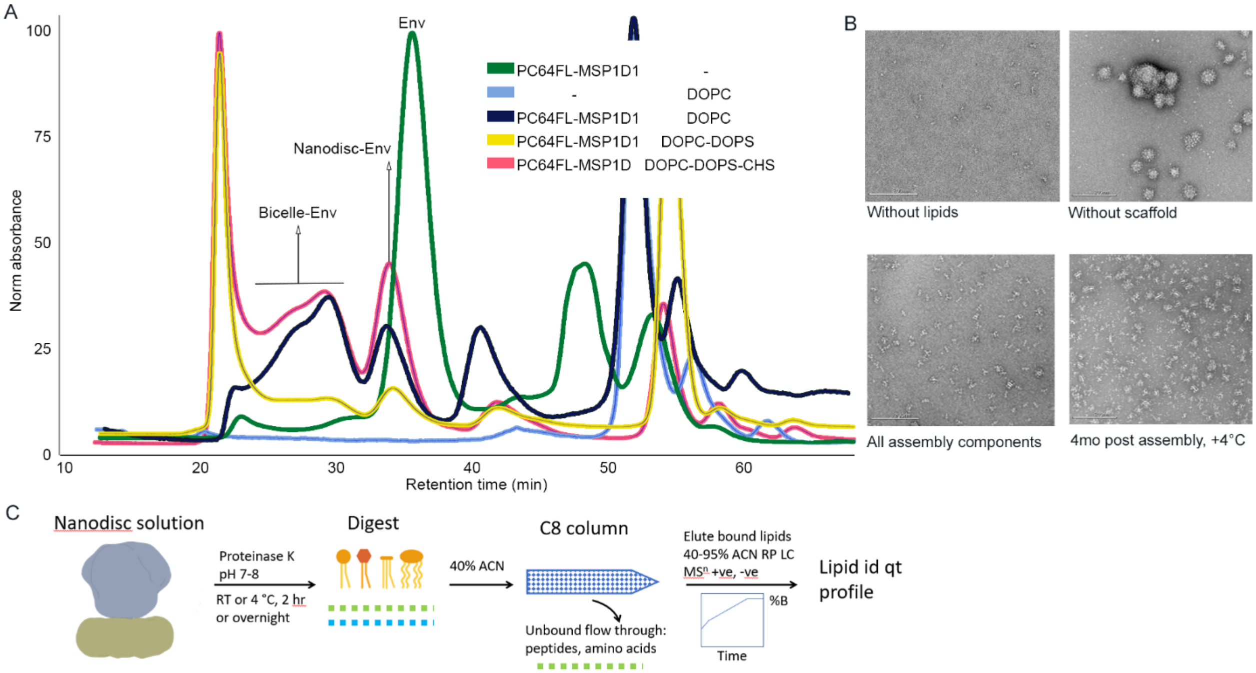

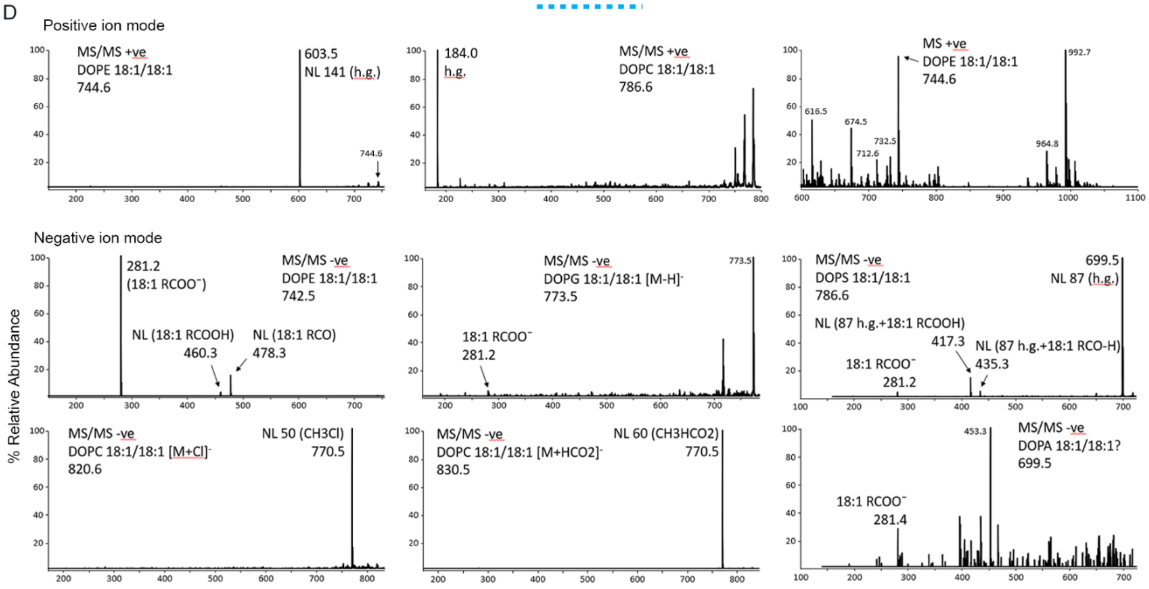

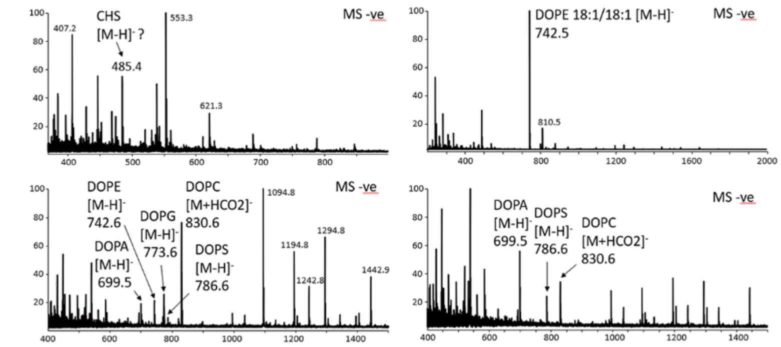

E

Identified lipids in nanodisc	Ion form	Exact mass m/z	MS/MS id signature	Ionization mode
**DOPE 18:1/18:1**	[M+H]+	**744.5538**	NL 141	positive
**DOPC 18:1/18:1**	[M+H]+	**786.6007**	184	positive
CHS	[M+Na]+	509.3607	not detected	positive (MS1)
DOPA 18:1/18:1	[M-H]-	699.4970	281	negative (MS1)
**DOPE 18:1/18:1**	[M-H]-	**742.5392**	281, NL 282, NL 264	negative
**DOPS 18:1/18:1**	[M-H]-	**786.5291**	NL 87	negative
**DOPG 18:1/18:1**	[M-H]-	**773.5338**	281	negative
**DOPC 18:1/18:1**	[M+Cl]-	**820.5624**	NL 50 (CH3Cl)	negative
	[M+HCO2]-	**830.5911**	NL 60 (CH3HCO2)	negative
PIP2 18:1	[M-H]-	1021.4825	not detected	negative
	[M-2H]2-	510.2376	not detected	negative
CHS	[M-H]-	485.3631	not detected	negative (MS1)

Figure S1 1. Controls for lipid assembly. Related to figure 1. A) Representative size exclusion chromatograms from a lipid mixture composition screen. B) representative raw micrographs of control reactions in the absence of scaffold or lipids and when all assembly components were present with an example of discs stored for 4 months at +4°C. C) Digest and conquer lipid analysis workflow. D) Selected disc lipid spectra in positive and negative ion mode. E) Disc lipid identification summary table. NL, neutral loss; h.g., head group; bold font, MS/MS confirmed.

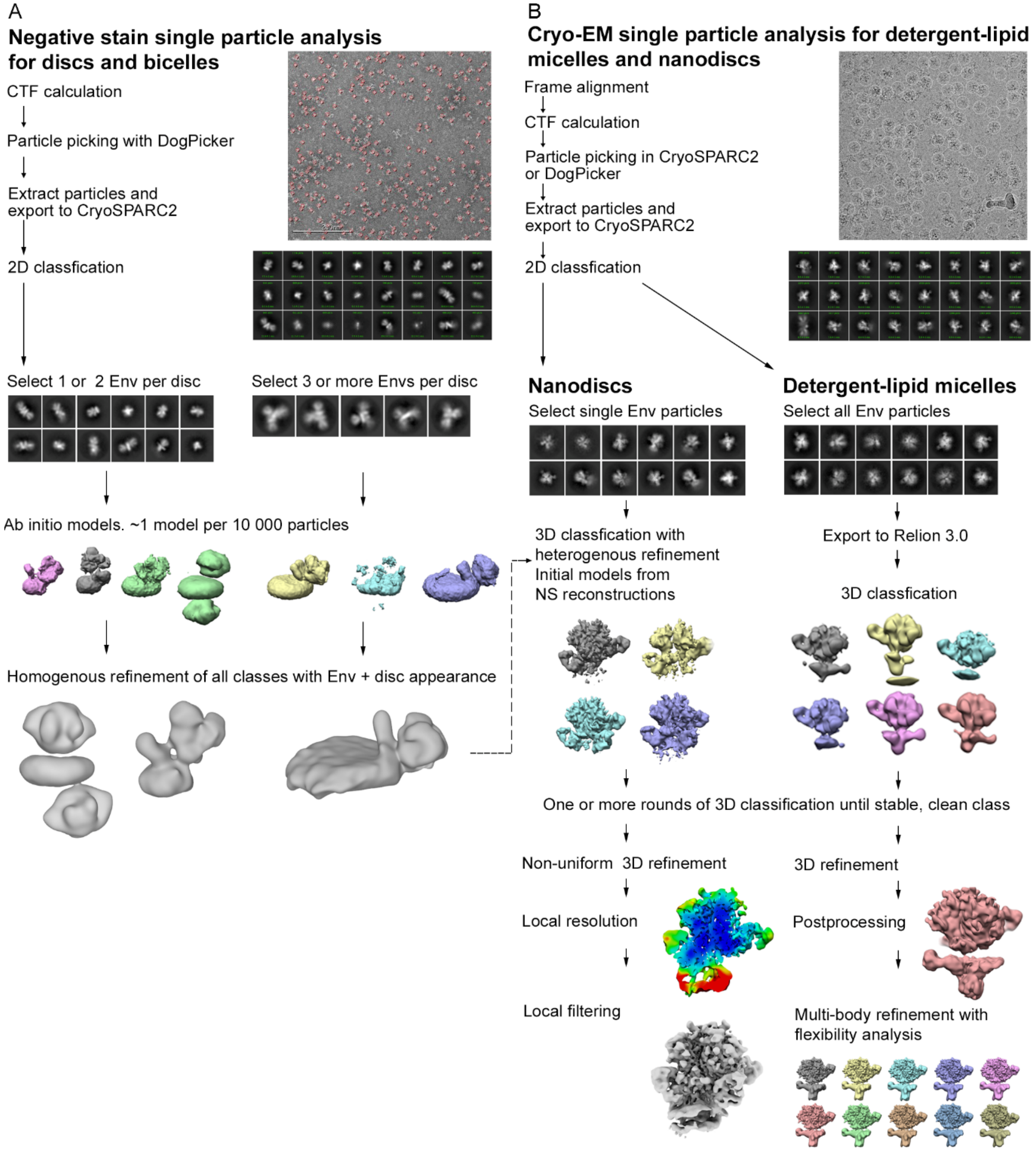

Figure S2. Single particle data processing workflows for analyzing different assemblies. Related to figures 1, 2, 3 and 4. A) negative stain EM, and B) cryo-EM.

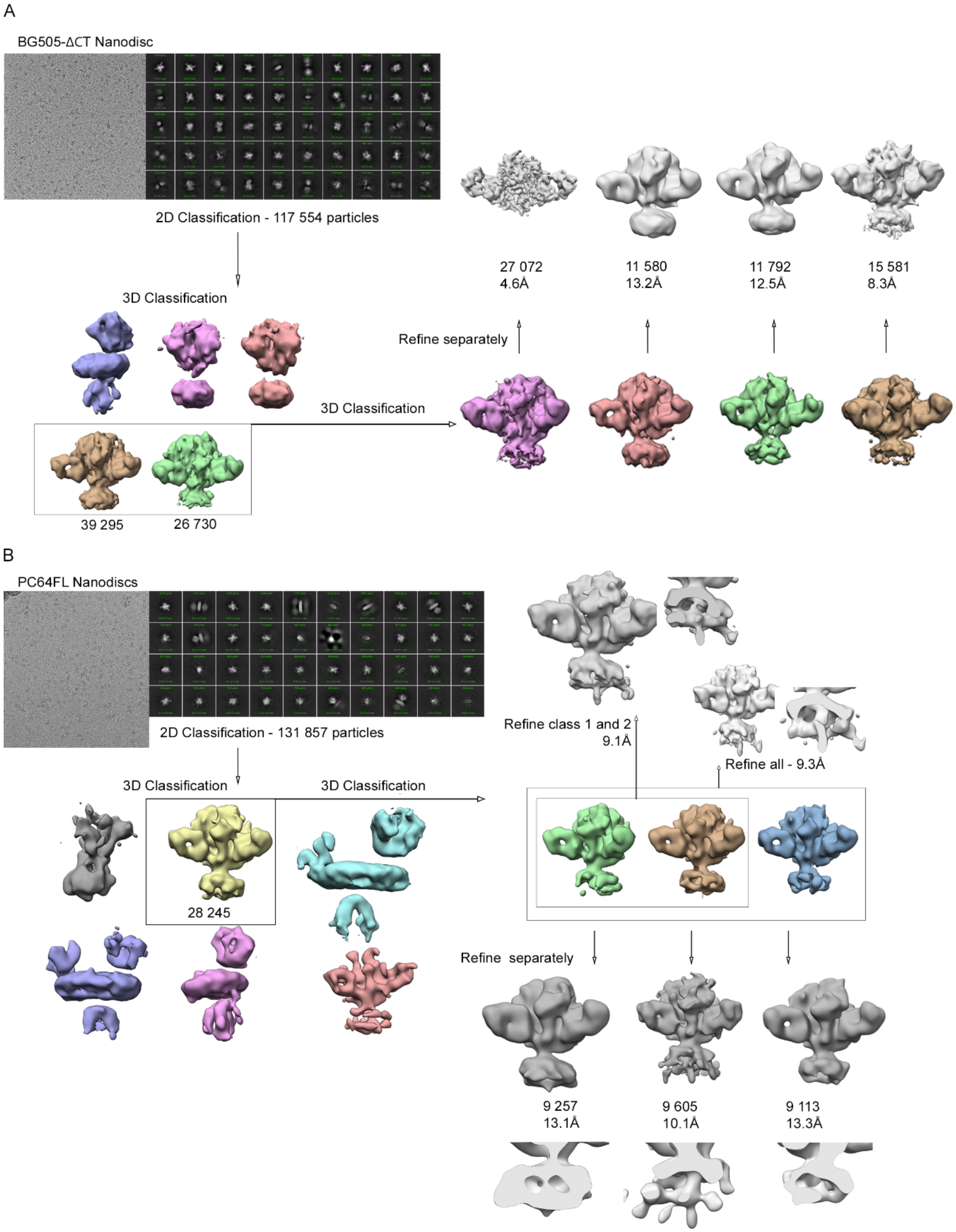

Figure S3. Representative raw micrographs and data processing workflows. Related to figure 2. A) BG505ΔCT and B) PC64FL nanodiscs. 3D classes with features of PGT151 stabilized ectodomain were selected for further 3D classification and refinement. In PC64FL embedded in nanodiscs, the additional membrane-embedded density was confirmed by refining the subclasses of nanodiscs independently.

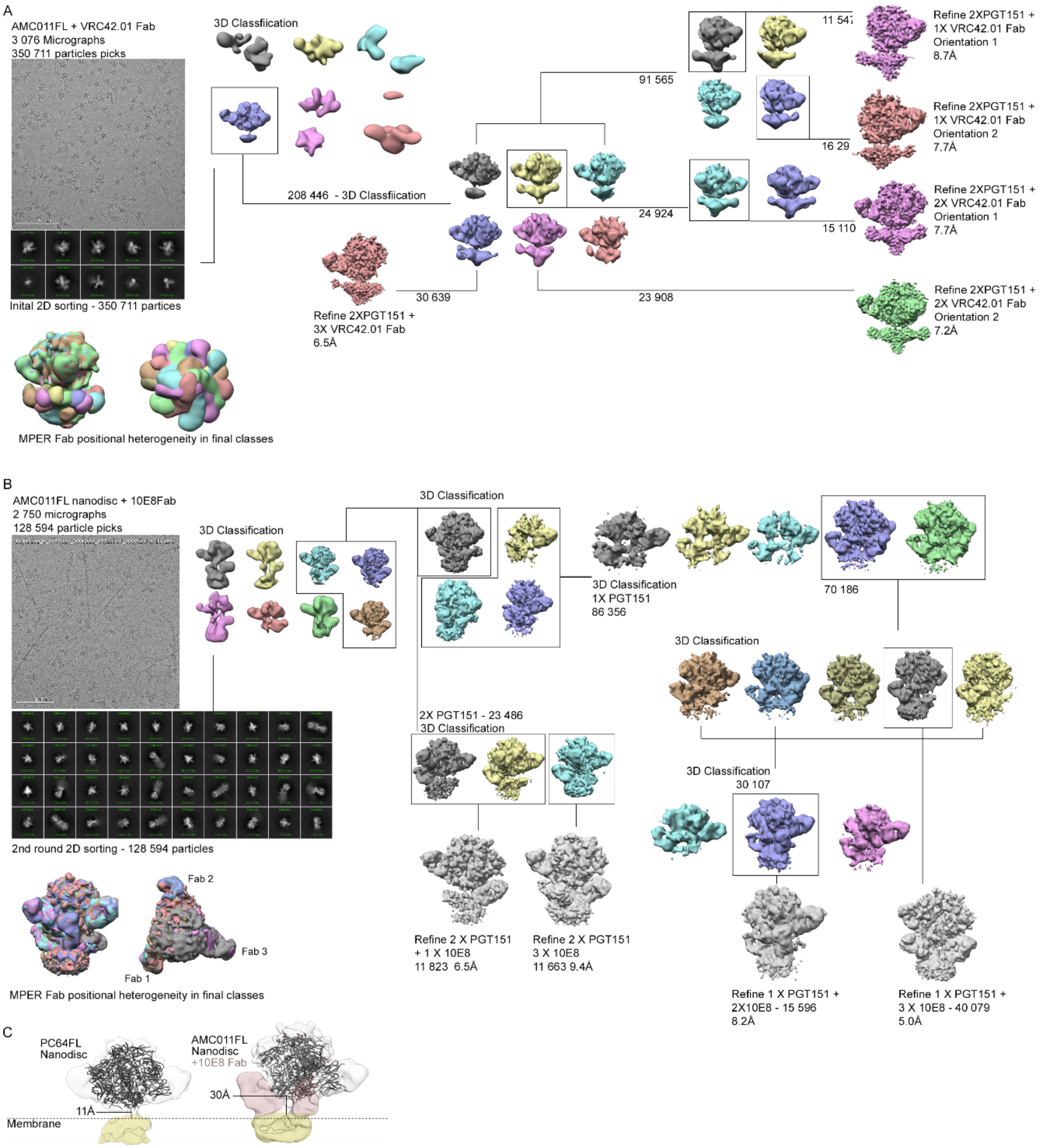

Figure S4. Comparison of data processing workflows. Related to figures 3 and 4. A) Detergent-lipid micelle approach and B) nanodisc approach. In the micelle approach, the MPER showed significant positional heterogeneity due to the flexible micelle and leading to near-continuous distribution of Fab positions. This, in combination with compositional heterogeneity originating from different Fab occupancy states led to significant reduction of particles in final, stable classes. In the nanodisc approach, the disc provides additional support for the Fab leading to three distinct and fixed MPER Fab positions thus reducing the heterogeneity and number of discarded particles. Classification for both approaches were tested in Relion 3.0 and CryoSPARC2. In the micelle samples, different Fab occupancy classes could not be efficiently separated by CryoSPARC2 heterogenous refinement whereas, with the nanodisc approach, both software provided comparable Fab occupancy separation. C) Comparison of ectodomain height in PC64FL nanodisc without MPER Fabs and AMC011FL nanodisc with 10E8 Fab. Height from the membrane surface is indicated.

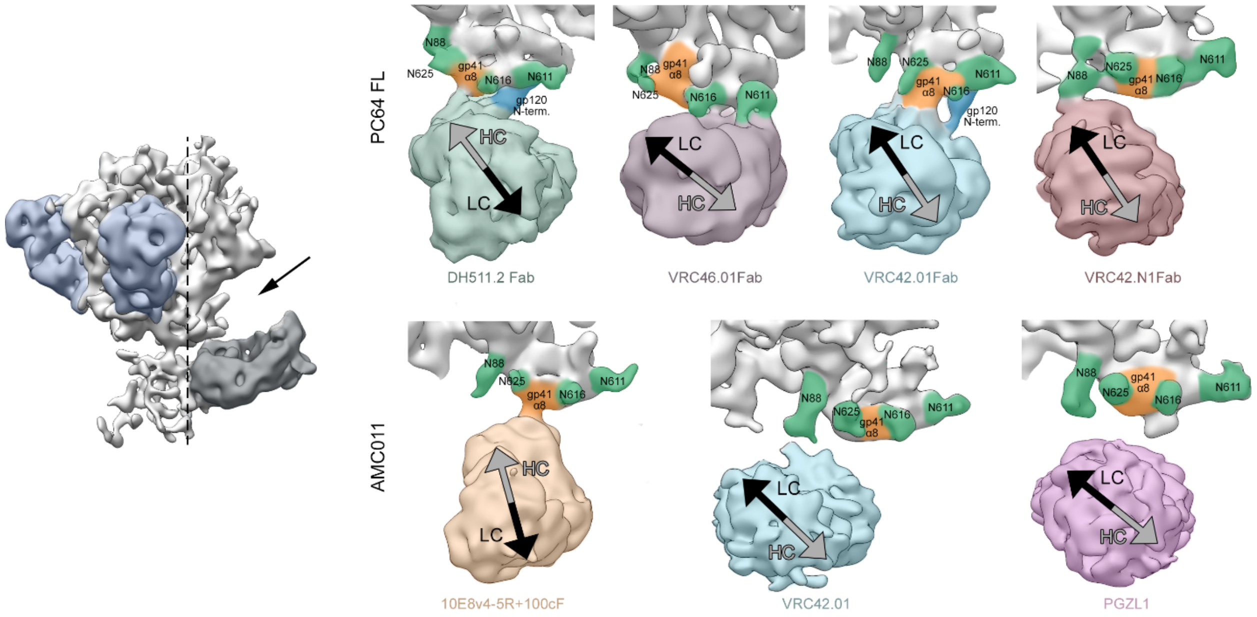

Figure S5. Proximity and positioning of MPER targeting Fabs in detergent-lipid micelle samples in relation to ectodomain. Related to figure 3. Densities corresponding to glycans, α8 helix and gp120 N-terminus are highlighted. Orientation of heavy (HC) and light chain (LC) are also indicated.

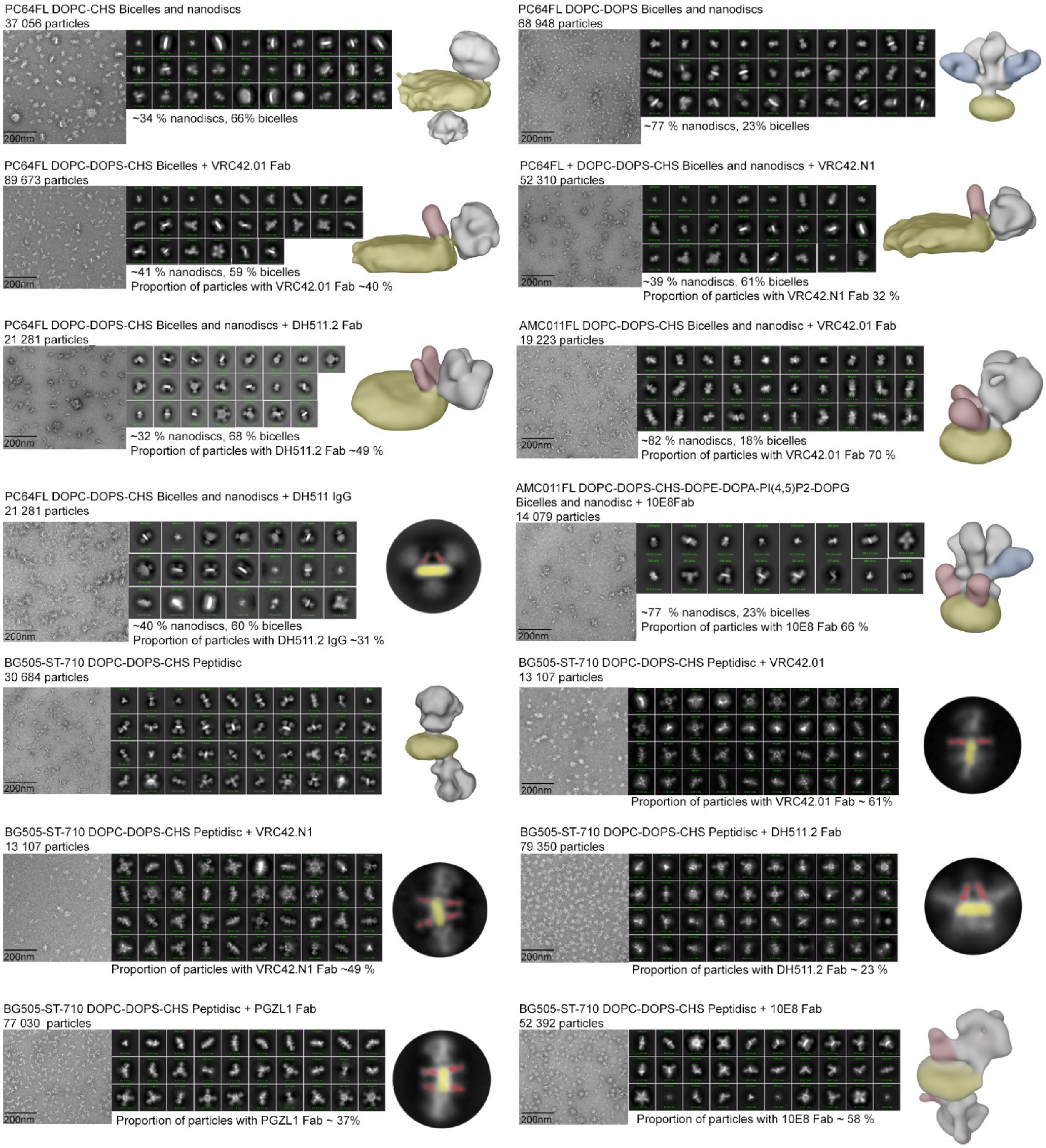

Figure S6. A panel of additional complexes analyzed as controls and as part of the method development. Related to figures 1, 2 and 4. Where data processing did not yield 3D reconstructions, matching features observed in 2D classes and representative 2D classes are shown. PGT151 Fab is highlighted in blue, MPER targeting Fab in red and the lipid bilayer in yellow. Proportion of nanodiscs and bicelles was estimated based on number of particles in 2D classes so that one and two Env particle classes with smaller diameter disc were counted as nanodiscs and the rest as bicelles. Proportion of MPER Fab bound particle was estimated based on particle number of combined 2D classes that have MPER Fab features for each dataset.

Table S1. Related to figures 3, 4 and 5. Summary of EM samples and imaging conditions.

**Sample Name**	Type of assembly	NS / Cryo	Microscope	Number of raw micrographs	Total number of Env particles	Number of particles used for reconstructions	Resolution (Å)	EMDB/PDB
PC64FL + PGT151 Fab + VRC42.01 Fab	Micelle	Cryo	Krios	8576	479 362	43 834	7.4	21321
AMC011FL + PGT151 Fab + VRC42.01 Fab	Micelle	Cryo	Krios	3078	350 711	23 908	7.2	21322
PC64FL + PGT151 Fab + VRC42.N1 Fab	Micelle	Cryo	Krios	2457	491 727	5 809	8.6	21323
PC64FL + PGT151 Fab + DH511.2 Fab	Micelle	Cryo	Krios	4463	748 662	7 661	7.5	21324
PC64FL + PGT151 Fab + VRC46.01 Fab	Micelle	Cryo	Krios	2288	340 716	30 378	9	21326
AMC011FL + PGT151 Fab + PGZL1 Fab	Micelle	Cryo	Krios	5788	279 853	21 264	6.6	21327
AMC011FL + PGT151 Fab + 10E8v4-5R 100cF Fab	Micelle	Cryo	Krios	6751	599 629	23 653	7.8	21328
BG505delCT + PGT151 Fab	Nanodisc	Cryo	Arctica	2343	117 554	13 324	9.9	21329
BG505delCT (Ectodomain) + PGT151 Fab	Nanodisc	Cryo	Arctica	2343	117 554	27 027	4.6	21330
PC64FL + PGT151 Fab	Nanodisc	Cryo	Arctica	3909	131 857	19 132	9.1	21331
AMC011FL + 2 X PGT151 Fab + 1 X 10E8 Fab	Nanodisc	Cryo	Arctica	2750	128 594	11 823	6.5	21332
AMC011FL + 2 X PGT151 Fab + 3 X 10E8 Fab	Nanodisc	Cryo	Arctica	2750	128 594	11 663	9.4	21333
AMC011FL + 1 X PGT151 Fab + 2 X 10E8 Fab	Nanodisc	Cryo	Arctica	2750	128 594	15 596	8.2	21334
AMC011FL + 1 X PGT151 Fab + 3 X 10E8 Fab	Nanodisc	Cryo	Arctica	2750	128 594	40 079	5	21335/6VPX
PC64FL	Bicelle	NS	Spirit	273	37 056	9 219	19	21336
PC64FL + VRC42.01 Fab	Bicelle	NS	Spirit	515	89 673	29 792	18	21337
PC64FL + DH511.2 Fab	Bicelle	NS	Spirit	440	21 281	11 154	20	21338
PC64FL + PGT151 Fab	Nanodisc	NS	Spirit	147	68 948	18 963	18	21339
PC64FL + VRC42.N1 Fab	Bicelle	NS	Spirit	417	52 310	23 785	17	21340
AMC011FL + VRC42.01 Fab	Nanodisc	NS	Spirit	106	19 223	5 004	19	21341
AMC011FL + 10E8 Fab	Nanodisc	NS	Spirit	240	14 079	4 172	26	21342
BG505-ST-710	Peptidisc	NS	Spirit	220	30 684	4 156	18	21343
BG505-ST-710 + 10E8 Fab	Peptidisc	NS	Spirit	403	52 392	17 567	20	21344
