# Supplementary material for: HIV-1 Envelope and MPER Antibody Structures in Lipid Assemblies
Source: Cell Rep. 2020 Apr 28;31(4):107583. doi: 10.1016/j.celrep.2020.107583 (PMC7196886; doi:10.1016/j.celrep.2020.107583)
Supplement: Document S2. Article plus Supplemental Information [file mmc3.pdf]

Cell Reports

Article

HIV-1 Envelope and MPER Antibody Structures in Lipid Assemblies

Graphical Abstract

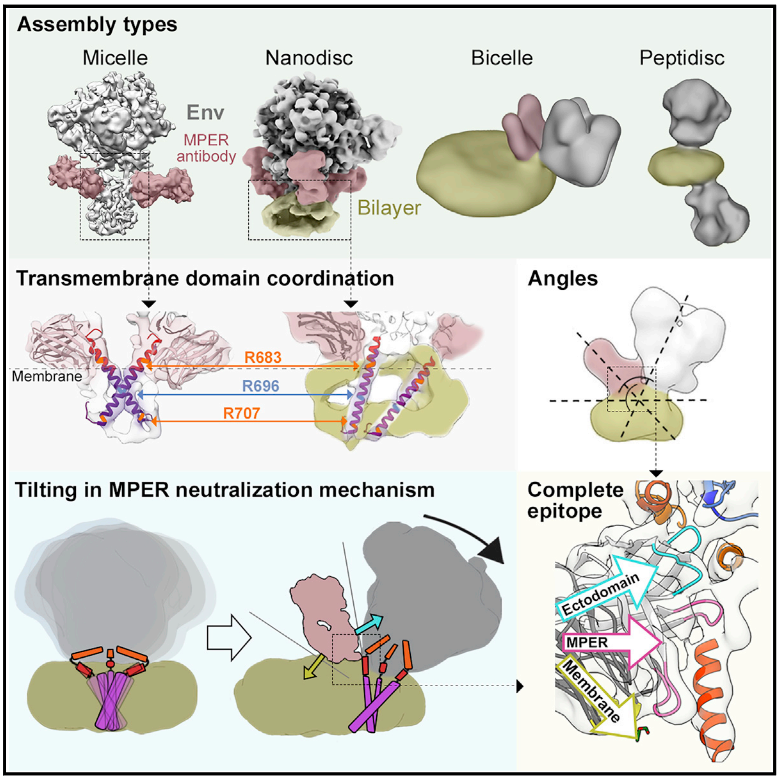

Highlights

- Full-length, wild-type Env incorporated into different lipid assemblies
- Structural studies of MPER antibody quaternary epitope in bilayer
- Structures reveal a tilting component in the MPER antibody binding mechanism
- Assembly platform can be adapted to vaccine design purposes

Authors

Kimmo Rantalainen, Zachary T. Berndsen, Aleksandar Antanasijevic, ..., John R. Yates III, William R. Schief, Andrew B. Ward

Correspondence

andrew@scripps.edu

In Brief

Rantalainen et al. explore approaches to assemble HIV envelope glycoprotein into lipid assemblies, creating a more native environment for structural studies of MPER targeting antibodies. Results illustrate the dynamics of the glycoprotein and show that, in these assemblies, MPER targeting antibodies tilt the glycoprotein in relation to the bilayer.

Rantalainen et al., 2020, Cell Reports *31*, 107583
April 28, 2020 © 2020 The Author(s).
https://doi.org/10.1016/j.celrep.2020.107583

CellPress

Cell Reports

CellPress
OPEN ACCESS

Article

HIV-1 Envelope and MPER Antibody Structures in Lipid Assemblies

Kimmo Rantalainen,[1,2] Zachary T. Berndsen,[1,2,10] Aleksandar Antanasijevic,[1,10] Torben Schiffner,[3,4] Xi Zhang,[5] Wen-Hsin Lee,[1,2] Jonathan L. Torres,[1,2] Lei Zhang,[4] Adriana Irimia,[1,2,3] Jeffrey Copps,[1,2] Kenneth H. Zhou,[6] Young D. Kwon,[6] William H. Law,[6] Chaim A. Schramm,[6] Raffaello Verardi,[6] Shelly J. Krebs,[7] Peter D. Kwong,[6] Nicole A. Doria-Rose,[6] Ian A. Wilson,[1,2,3,8] Michael B. Zwick,[4] John R. Yates III,[5] William R. Schief,[2,3,4,9] and Andrew B. Ward[1,2,3,11,*]

[1]Department of Integrative Structural and Computational Biology, The Scripps Research Institute, La Jolla, CA, 92037, USA
[2]International AIDS Vaccine Initiative Neutralizing Antibody Center, The Scripps Research Institute, La Jolla, CA 92037, USA
[3]Center for HIV/AIDS Vaccine Development, The Scripps Research Institute, La Jolla, CA 92037, USA
[4]Department of Immunology and Microbiology, The Scripps Research Institute, La Jolla, CA 92037, USA
[5]Department of Chemical Physiology, The Scripps Research Institute, La Jolla, CA, USA
[6]Vaccine Research Center, National Institute of Allergy and Infectious Diseases, NIH, Bethesda, MD 20892, USA
[7]U.S. Military HIV Research Program, Walter Reed Army Institute of Research, Silver Spring, MD 20910, USA
[8]Skaggs Institute for Chemical Biology, The Scripps Research Institute, La Jolla, CA 92037, USA
[9]Ragon Institute of MGH, MIT, and Harvard, Cambridge, MA 02129, USA
[10]These authors contributed equally
[11]Lead Contact
*Correspondence: andrew@scripps.edu
https://doi.org/10.1016/j.celrep.2020.107583

SUMMARY

Structural and functional studies of HIV envelope glycoprotein (Env) as a transmembrane protein have long been complicated by challenges associated with inherent flexibility of the molecule and the membrane-embedded hydrophobic regions. Here, we present approaches for incorporating full-length, wild-type HIV-1 Env, as well as C-terminally truncated and stabilized versions, into lipid assemblies, providing a modular platform for Env structural studies by single particle electron microscopy. We reconstitute a full-length Env clone into a nanodisc, complex it with a membrane-proximal external region (MPER) targeting antibody 10E8, and structurally define the full quaternary epitope of 10E8 consisting of lipid, MPER, and ectodomain contacts. By aligning this and other Env-MPER antibody complex reconstructions with the lipid bilayer, we observe evidence of Env tilting as part of the neutralization mechanism for MPER-targeting antibodies. We also adapt the platform toward vaccine design purposes by introducing stabilizing mutations that allow purification of unliganded Env with a peptidisc scaffold.

INTRODUCTION

HIV envelope glycoprotein (Env) is a homotrimeric transmembrane protein belonging to the class I viral fusion proteins. Binding of Env to host receptor CD4 and coreceptors CCR5 or CXCR4 leads to a cascade of conformational changes and eventually virus entry. Each of the three Env protomers are linked through a membrane-proximal external region (MPER) to a single-pass transmembrane domain (TMD) and an intracellular C-terminal domain (CTD), which play critical roles in fusion (Chen, 2019; Harrison, 2015; Santos da Silva et al., 2013). While several structures of isolated ectodomains, MPER peptides and TMDs have been determined using X-ray crystallography, cryoelectron microscopy (cryo-EM), and nuclear magnetic resonance (NMR), structural studies of the complete Env have been complicated by the challenging nature of the trimer (Chen, 2019; Ward and Wilson, 2017). Low expression levels and poor long-term stability of native Env, together with structural flexibility and shedding of gp120 subunit (Hammonds et al., 2003), have led structural biologists to stabilize the trimer with mutations and/or antibodies so as to achieve higher resolution details. Structural intermediates have been captured after receptor binding and in complex with antibodies, which illustrate the intricately coordinated structural transitions of Env (Lu et al., 2019; Ozorowski et al., 2017; Tran et al., 2012). This flexibility is compounded when parts below the ectodomain are included, so structures of CTD, TMD, and unliganded MPER have only been resolved in isolation using NMR (Chiliveri et al., 2018; Dev et al., 2016; Kwon et al., 2018a; Sun et al., 2008). In these studies, the MPER is found as a membrane-embedded amphipathic helix and trimeric protrusion from the membrane, the TMD as a three-helix bundle or separate tilted helix, and the CTD as an elongated set of three amphipathic helices, leaving open questions as to how these conformations relate to the full Env trimer assembly. These structures have however provided important insights in the dynamic nature of these domains and show the

This is an open access article under the CC BY license (http://creativecommons.org/licenses/by/4.0/).

capacity to adapt to different stages of the entry and fusion process akin to those observed in the ectodomain. Structures of PGT151-antibody-stabilized ectodomains from C-terminally truncated JRFL, BG505, and B41 Env constructs and wild-type, full-length (FL) Env from PC64 and AMC011 donors confirmed the structural similarity to stabilized soluble Env, but due to structural flexibility in micelle-embedded domains, the MPER, TMD, and CTD have remained unresolved (Cao et al., 2018; Lee et al., 2016; Rantalainen et al., 2018; Torrents de la Peña et al., 2019).

A multitude of broadly neutralizing antibodies (bNAbs) have now been characterized, targeting various sites on HIV-1 Env (Haynes and Mascola, 2017; Sok and Burton, 2018). Structures of these bNAbs in complex with Env have paved the way for structure-based vaccine design by facilitating the identification of stabilizing mutations that allow large-scale expression and purification of the unliganded ectodomain (Dey et al., 2018; Guenaga et al., 2017; Joyce et al., 2017; Klein et al., 2013; Kulp et al., 2017; Kwon et al., 2015; Ringe et al., 2017; Rutten et al., 2018; Schoofs et al., 2019; Sliepen et al., 2019; Torrents de la Peña and Sanders, 2018; Ward and Wilson, 2017; Yuan et al., 2019). The epitopes of MPER-targeting bNAbs are missing from almost all of these Envs to increase solubility and stability and, therefore, remain the least understood bNAb epitope. Well-known members of the MPER bNAb family are antibodies 10E8 and 4E10, which share common features and high neutralization breadth over different HIV strains (Cardoso et al., 2005; Huang et al., 2012; Stiegler et al., 2001; Zwick et al., 2001). More recently, a new MPER targeting antibody lineage PGZL1 from donor PG13 and three lineages from donor RV-217 (VRC42, VRC43, and VRC46) with outstanding breadth were discovered. These studies describe in more detail the maturation of MPER-targeting antibodies (Krebs et al., 2019; Zhang et al., 2019). While the mature PGZL1 neutralized 84% of the 130-virus panel with ~21% heavy chain (HC) and ~13% light chain (LC) somatic hypermutation (SHM) at the nucleotide level, the recombinant sublineage variant H4K3 (17% HC, 12% LC SHM) was able to neutralize 100% of the panel, and importantly, the germline-reverted variant neutralized 12% of the panel. In RV-217, all three lineages matured with lower SHM (9%–13%) with up to 96% neutralization breadth, whereas the VRC42 lineage reached 50% neutralization breadth with only 2% SHM. These impressive bNAbs, together with the high conservation of MPER sequence, have stimulated renewed interest in MPER-targeting vaccine design and the use of MPER antibodies for post-exposure prophylaxis. Crystal structures of many MPER Fabs have been solved, alone and in complex with MPER peptide and/or with additional short-tailed lipid headgroups (Irimia et al., 2016, 2017; Krebs et al., 2019; Williams et al., 2017; Zhang et al., 2019), providing valuable details of MPER peptide and membrane lipid recognition. For example, in the case of 10E8, residues in CDRL1 and CDRH3 were shown to bind to phosphatidic acid and phosphatidylglycerol headgroups. The structure of the most recently discovered MPER antibody, LN01, was resolved in complex with MPER peptide and full TMD, revealing a straight, continuous MPER-TMD helix and a second conformation of the helix with a kink at conserved G691 (Pinto et al., 2019). Two intermediate resolution cryo-EM studies of MPER-targeting antibodies (10E8 and PGZL1) in the context of the trimeric ectodomain provide insights into antibody approach angle, steric obstruction by glycans, and antibody-induced lifting of Env from the lipid surface (Lee et al., 2016; Zhang et al., 2019). Despite efforts to better understand MPER antibodies, the full quaternary epitope has remained elusive, emphasizing the need to study structures of these antibodies in more native environments.

Reconstitution of membrane proteins into lipid nanodiscs in combination with recent advances in cryo-EM has shown great promise as a tool for structural biology of membrane proteins (Denisov and Sligar, 2016; Efremov et al., 2017). This method was made possible by the introduction of apolipoprotein A based scaffold proteins and associated nanodisc assembly methodology in the early 2000s (Bayburt et al., 2002). Since then several other scaffold types have been introduced, all of which facilitate spontaneous assembly of the target protein into lipid bilayer discs upon detergent removal. In addition to rendering hydrophobic and transmembrane molecules to be essentially as manageable as soluble proteins, the nanodisc technology offers exceptional versatility for experimental design, allowing different disc diameters and lipid compositions to be co-assembled with the target molecule.

In this work, we present approaches to study HIV Env in membranous environments by assembling FL, wild-type Envs from PC64 (clade A) and AMC011 (clade B) donors and a C-terminally truncated BG505 Env (clade A) into detergent-lipid micelles, bicelles, and nanodiscs. In combination with single-particle EM analysis, these protein-lipid assemblies provide tools for studying HIV Env in a lipid bilayer as well as the binding mechanism of MPER bNAbs. In the lipid bilayer systems, MPER bNAbs induce tilting of Env relative to the membrane surface, forming a wedge between the ectodomain and the lipid surface. In addition, by complexing the AMC011FL nanodisc with 10E8 Fab, we were able to characterize the full tripartite quaternary epitope consisting of lipid, peptide, and glycan contacts. Finally, we show that this methodology can be adapted for vaccine engineering by introducing stabilizing mutations into a C-terminally truncated BG505 construct, allowing presentation of the full array of bNAb epitopes, including MPER, without the need for a stabilizing antibody.

RESULTS

Env Incorporation into Detergent-Lipid Micelles, Bicelles, and Nanodiscs

Different assembly pathways were experimentally assessed to establish a modular platform for studying Env and the neutralization mechanism of MPER antibodies in lipid environments (Figures 1 and S1). To build upon the detergent-lipid micelle approach described earlier (Blattner et al., 2014; Lee et al., 2016; Rantalainen et al., 2018; Torrents de la Peña et al., 2019), through a more complete detergent removal and addition of scaffold proteins MSP1D1 or peptidisc, Env could be incorporated into bicelles and nanodiscs with various lipid compositions (Figure 2). In the detergent-lipid micelle approach, lipid molecules are exchanged by partial detergent

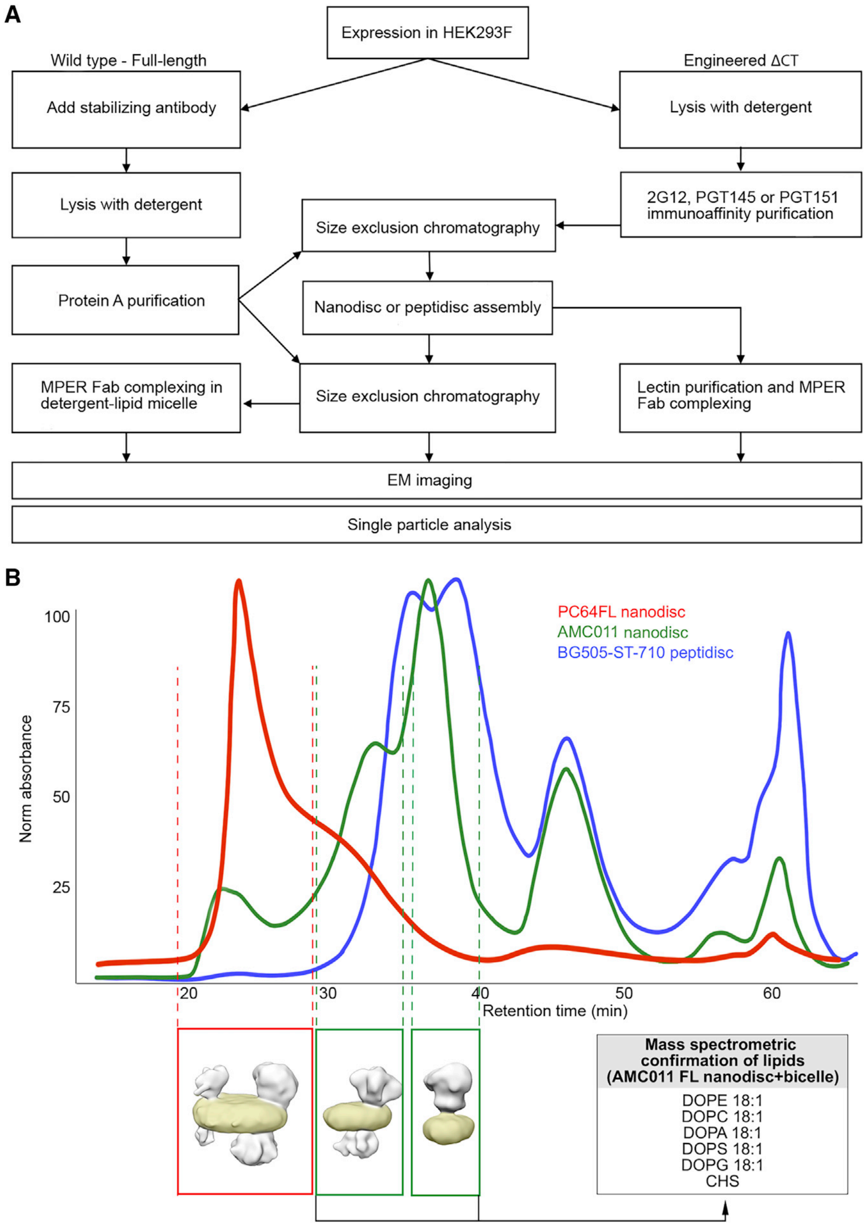

Figure 1. Preparation of Env Lipid Assemblies

(A) Overview of the workflow used to generate and analyze different lipid assemblies.

(B) Typical size-exclusion chromatograms of different Env assemblies. 3D reconstructions below the chromatogram peaks illustrate the corresponding forms of the assembly. The lipid bilayer is highlighted in yellow. Incorporation of lipids was confirmed by mass spectrometry from a pool of AMC011 FL nanodiscs and bicelles.

See also Figures S1, S2, and S6.

removal, leading to complexes that are unstable (~1–2 days) but suitable for high-resolution determination of the ectodomain by cryo-EM. In nanodiscs, a complete detergent removal is done over ~48 h in the presence of MSP1D1 scaffold protein, leading to a stable lipid bilayer encircled by the scaffold. The peptidisc approach follows the same principles, but the scaffold is now a short, bi-helical, engineered peptide (Carlson et al., 2018). In the lipid bicelle approach, similar detergent removal leads to heterogeneously sized bicelles capped by lipid molecules and/or scaffold. Formation of bicelles versus nanodiscs was concluded to be largely dependent on the Env clone. FL PC64 Env (PC64FL) Env preferred the formation of larger bicelle assemblies with multiple Envs compared to AMC011FL, BG505ΔCT, and BG505-ST-710 (Figures 1B and S6), which predominantly resulted in smaller discs with one or two Envs. We assume this to result from differences in how the hydrophobic TMD and CTD of the different constructs interact with the lipids and scaffold protein during assembly. Nanodisc scaffold MSP1D1 resulted in ~10-nm-diameter discs, whereas bicelle size is less restricted and varied from ~15 to ~25 nm. Therefore, assemblies with a diameter of 10 nm were considered as nanodiscs and larger assemblies as bicelles (Figures 2B–2D). Selection of lipids also affected the size distribution of the assemblies, although we could not systematically define the effect of lipid composition (Figures S1A and S6). Env occupancy varied from one to two in nanodiscs to between two and four in larger bicelles. Size exclusion chromatography did not efficiently separate the different species, and in most samples, nanodiscs and bicelles were pooled. Different Env occupancies could, however, be easily separated computationally during 2D and 3D classification in EM data processing (Figure S2). Incorporation of lipid molecules was confirmed with mass spectrometry, where five out of seven added lipid types could be confirmed (Figures S1C, S1D, and S1E). The Env incorporation and recovery ratio varied between 5% and 50%. Incorporation was noted to be more efficient and reproducible in smaller, 50- to 100-μL reaction volumes. In control reactions without scaffold protein, less stable proteoliposomes were formed (Figure S1B). In the absence of lipids and scaffold, rosettes of two or more Envs were formed, leading to aggregation after prolonged incubation (~1–2 days; Figure S1B). PC64FL and AMC011FL Env nanodiscs and bicelles remained intact at +4°C for up to 4 months but could not be recovered after freezing or complete dehydration (Figure S1B). Samples were primarily assessed using negative-stain and cryo-EM single-particle image analysis (Figure S2).

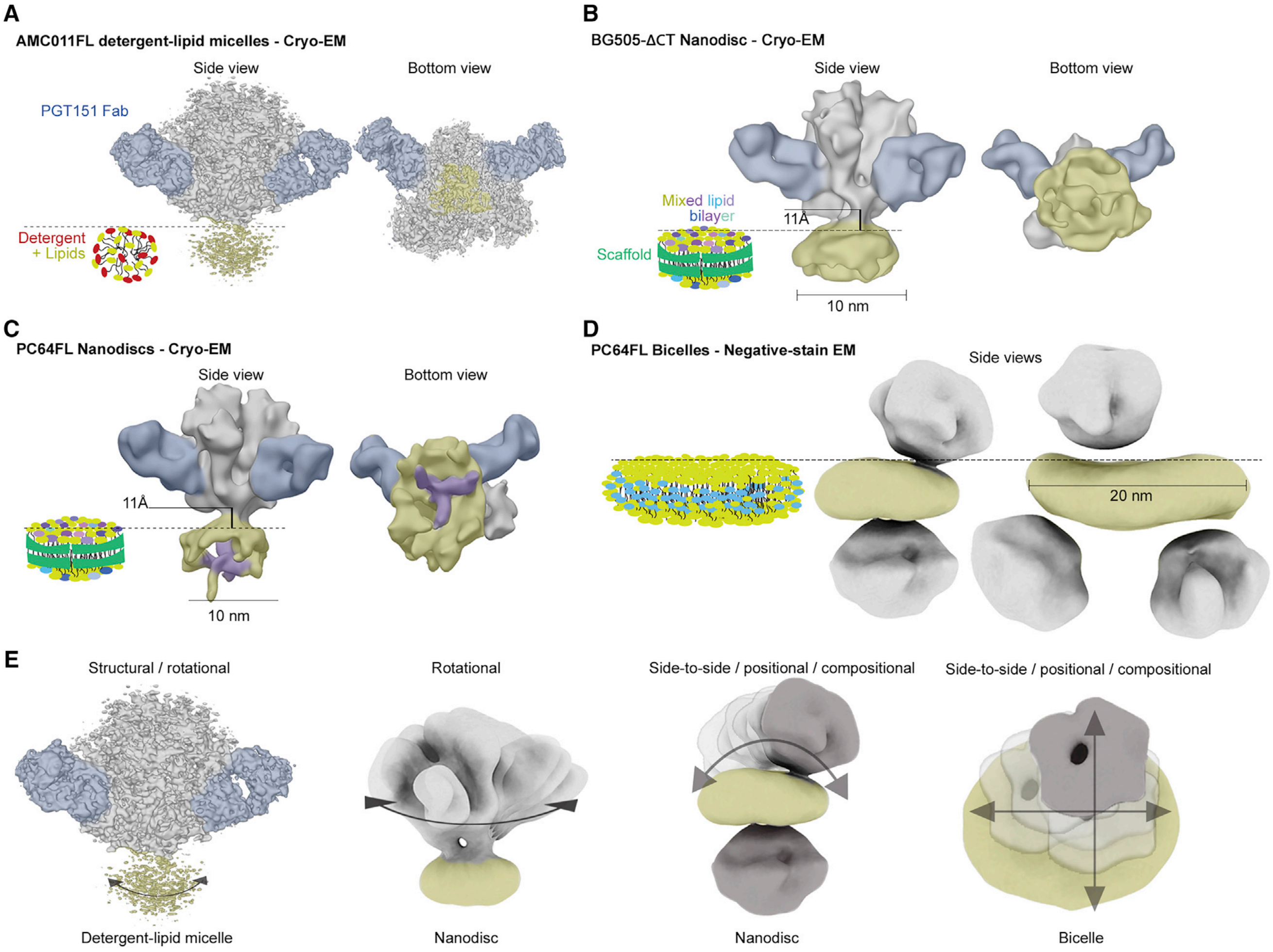

Figure 2. Comparison of Different Sample Preparation Approaches and Major Components of Sample Heterogeneity

Stabilizing PGT151 Fab highlighted in blue. Different assembly types are presented with cartoons next to negative stain and cryo-EM 3D reconstructions. The Env ectodomain is highlighted in gray, PGT151 in blue, and the micelle and lipid bilayer in yellow.

(A) The ectodomain could be reconstructed to 3–5 Å resolution from all lipid-detergent micelle samples.

(B and C) When the whole nanodisc assembly was reconstructed from cryo-EM data, global resolution remained at ~9 Å, but now the height from the bilayer surface could be measured and is indicated for PC64FL (B) and BG505ΔCT (C). Height was estimated by fitting an Env model to the ectodomain density and a modeled lipid bilayer patch in the EM density corresponding to the disc. The distance between the stable ectodomain ending at Asp664 and the closest atom of the bilayer is reported as the ectodomain height from the bilayer surface. Density that passes through the disc of PC64FL is highlighted in purple.

(D) Discs larger than the 10 nm in diameter defined by the MSP1D1 scaffold were considered as bicelles. In these, additional compositional heterogeneity limits the analysis to negative-stain EM reconstructions.

(E) Illustrative summary of the cumulative rotational, positional, and compositional heterogeneity indicated by the arrows and shading of Env in different assembly types.

See also Figures S2, S3, and S6.

Env Displays Several Degrees of Flexibility in Lipid Assemblies

In all detergent-lipid micelle PC64 and AMC011 FL samples, the PGT151-stabilized ectodomain could be reconstructed to 4–5 Å resolution as in our earlier studies (Figure 2A) (Rantalainen et al., 2018; Torrents de la Peña et al., 2019). In the nanodisc sample of BG505ΔCT, the PGT151-stabilized ectodomain was reconstructed to 4.6 Å resolution (Figure S3A), but the global resolution ranged between 9 and 12 Å when disc and regions below the ectodomain were included (Figure 2B; Table S1). Nonetheless, this resolution enabled positioning of the bilayer surface and measurement of stable part of ectodomain height from the membrane surface (ending at Asp664). In both BG505ΔCT and PC64FL, the ectodomain was 11 Å from the bilayer surface (Figures 2B and 2C). The PC64FL nanodisc reconstruction at 9 Å resolution contained a continuous density emanating from the bottom of the ectodomain and spanning the bilayer (Figures 2C and S3B). In bicelles, two to four FL Envs were incorporated, thereby greatly increasing sample heterogeneity and limiting the studies to negative-stain EM analysis (Figures 1B, 2D, S2A, and S6). In some 2D and 3D classes, bicelle curvature was also observed. In summary, Env assemblies showed several

degrees of rotational and positional flexibility as well as compositional heterogeneity in nanodiscs and bicelles, limiting high-resolution structure determination but likely reflecting the native flexibility of the trimer on the surface of membranes (Figure 2E). Taken that the isolated ectodomain could be reconstructed to higher resolution and that the bilayer was well defined in 2D and 3D classes, we concluded that HR2-MPER region at the intersection of the ectodomain and bilayer is the main contributor to the heterogeneity in the assemblies.

Env-MPER Fab Complexes in Detergent-Lipid Micelles Show Heterogenous Fab Positioning and TMDs Crossing at Residue R696

We next attempted to stabilize the flexible parts of Env and improve the epitope definition by addition of different MPER-targeting antibody Fabs in mixed detergent-lipid micelles (Figure 3). In all tested combinations of FL Env and MPER Fab, cryo-EM analysis showed no stable, high-resolution structural features for MPER, TMD, or CTD similarly to earlier cryo-EM studies (Figure 3A) (Lee et al., 2016; Rantalainen et al., 2018; Torrents de la Peña et al., 2019). One of the tested antibodies involved a variant of 10E8, called 10E8v4-5R+100cF, which was designed to have improved membrane-interaction capacity and showed 10-fold high neutralization potency (Kwon et al., 2018b). This antibody also failed to show stable, high-resolution structural features, suggesting that stable lipid headgroup contacts are not fully recovered in this approach. Despite collecting large cryo-EM datasets of over 1 million particles, the liganded complexes could not be refined beyond 6–9 Å resolution for the regions below the ectodomain due to structural and compositional heterogeneity. The flexibility was further emphasized with multibody 3D refinement of AMC011FL in complex with one copy of PGZL1 Fab (Video S1), where Fab and micelle show both horizontal and vertical movement in relation to the ectodomain. Similar evidence was provided by superimposition of different Fab occupancy classes from AMC011FL-VRC42.01 dataset showing a continuous Fab position around the micelle (Figure S4A). This positional heterogeneity of Fab, in addition to particle classes without MPER Fab significantly reduced the number of particles in final, stable classes. Regardless of the movement in relation to ectodomain, we were able to detect converging features, namely contacts to the α8-helix of gp41, the N terminus of gp120, proximity of glycans, and the orientation of the HC and LC of the antibody in relation to the ectodomain (Figure S5). By collecting a larger dataset and increasing the particle number of PC64FL in complex with VRC42.01 Fab, a subset of particles could be classified that contained continuous density from HR2 to MPER and into the TMD (Figure 3B; Table S1). Although the resolution did not enable *de novo* building of an atomic model, fitting in high-resolution structures of the complex components was sufficient to model the MPER and TMD. Interestingly, the individual transmembrane (TM) helices crossed the micelle in a tilted fashion forming an X shape with TMDs from adjacent protomers crossing at an $\sim$75° angle and at $\sim$50° in relation to the postulated membrane plane (Figure 3B). The helices crossed in the micelle at the conserved R696, a residue previously shown to be important for modulating conformational changes of the TMD (Cooper et al., 2018; Hollingsworth et al., 2018; Wang et al., 2019).

EM Analysis of MPER Antibodies Bound to Bicelle- and Nanodisc-Incorporated Env Reveals a Wedging and Tilting Component in the Binding Mechanism

We next analyzed the MPER antibody-binding mode in lipid bilayer assemblies with varied lipid content. In all tested combinations, addition of the Fab to Env embedded in a lipid bilayer (nanodisc or bicelle) led to displacement of Env to the side of the assembly and tilting of Env in relation to bilayer (Figures 4, 5, and S6). This suggested that Env tilting is a common phenomenon of MPER antibody binding and prompted us to align the complexes to the bilayer instead of the ectodomain. By comparing the bilayer assemblies to detergent-lipid reconstructions, we also noted that the angle between the ectodomain and the Fab is identical between the two assembly types (Figure 4B). To confirm the tilting component of MPER binding and further elucidate the tripartite quaternary epitope of the antibody, we then assembled AMC011FL in nanodiscs for cryo-EM analysis with a lipid mixture roughly following the lipid composition previously determined for HIV particles (Lorizate et al., 2013). We chose to complex the nanodisc with 10E8 Fab, as this is the most characterized MPER-targeting antibody. The complex was frozen on graphene oxide grids to improve particle orientation distribution for cryo-EM analysis. These improvements allowed collection of data with low concentration of sample ($\sim$0.1 mg/mL) and an adequate number of particles to classify stable particle subsets with different Fab occupancies from a relatively small dataset (128,594 particles; Figures 4C and S4B). Due to steric constraints introduced by the lipid bilayer, 10E8 now showed a fixed position in comparison to the detergent-lipid approach, which in turn improved the 3D classification accuracy of the particles (Figure S4B). When the reconstructions were aligned to a simulated bilayer model, the Env ectodomain with one and two 10E8 Fabs was $\sim$18 Å away and tilted 110° in relation to the bilayer surface, confirming the tilting observed with bicelles and negative-stain EM data (Figure 4D), whereas the third Fab led to an $\sim$10° more vertical Env orientation. The height of the stable part of Env ectodomain from the bilayer surface was now 30 Å compared to $\sim$18 Å with one or two Fabs (Figure S4C). In PC64FL and BG505ΔCT nanodiscs without MPER Fabs, this distance was 11 Å (Figures 2B and 2C). The highest global resolution ($\sim$5 Å) was obtained with the complex containing one copy of PGT151 Fab and three copies of 10E8 Fab, which also had the largest number of particles (Figure 4E). By fitting the crystal structure of 10E8 Fab together with AMC011 Env protomers in the reconstruction, we generated a hybrid model allowing the definition of the tripartite quaternary epitope and an estimate of the contacting residues (Figure 4E; Table 1; PDB: 6VPX). While local resolution estimations showed stabilization of the epitope between Fab A and Env up to 5 Å resolution, the disc, TMD, and CTD were less homogeneous (Figure 4F). The TMDs were, however, resolved as a continuous density, revealing that the TMDs bound to Fab A and B had straight TMD density tilted at 75° in relation to the bilayer surface. Fab C binding resulted in a TMD tilted at 50°, similar to micelle-embedded TMD in the PC64FL-VRC42.01 Fab complex (Figures 4G and 3B). The 10E8 Fabs bound to the AMC011FL nanodisc similarly had two distinct binding modes with different binding angles. Fab A and B had a more vertical orientation in relation

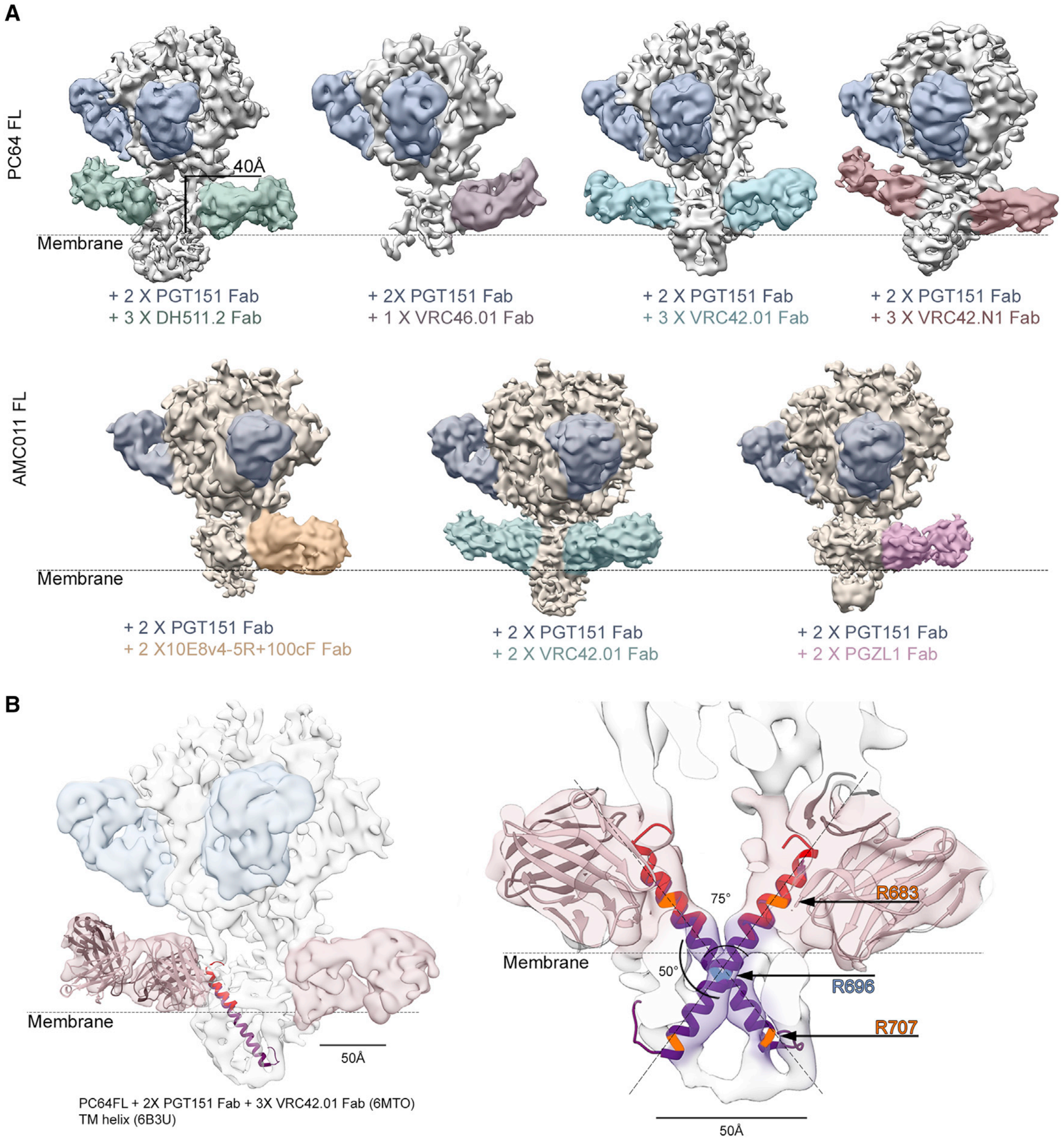

Figure 3. Cryo-EM Reconstructions of FL Env-MPER Fab Complexes in Detergent-Lipid Micelles

(A) PC64FL and AMC011FL Envs with a panel in of MPER-targeting Fabs showing variable Fab positions and occupancies. The estimated membrane position is indicated as well as the height of the structurally stable part of ectodomain (ending at Asp664) from the membrane surface. Membrane surface position in the absence of the bilayer is estimated based on MPER Fab position. Particle classes with one, two, and three Fabs could be classified from most of the datasets with all showing similar flexibility and heterogenous density in the micelle. When the second or third MPER Fab is not visible, it is bound behind the micelle, pointing away from the viewer.

(B) In the complex between PC64FL and VRC42.01 Fab, the MPER density could be followed through to the TMD as continuous density allowing docking of crystal structure of the Fab (PDB: 6MTO) and NMR structure of TMD helices (PDB: 6B3U). The position of R696 as the crossing point of the helices is indicated as well as residues R683 and R707 that are commonly positioned at the membrane boundaries.

See also Figures S2, S4, and S5 and Table S1.

to the ectodomain than Fab C, which was influenced by the proximal PGT151 (Figure 4H). Based on fitting of ectodomain and Fab structures in the map, contacts to the ectodomain that were also observed with detergent-lipid micelles (Figures 3 and S5) could now be better defined. Contacts to α8-helix (gp41) and gp120 by CDRH1 and HC framework region 3 (FR3) remained further apart in Fab C compared to Fab A and B (Figure 4E). As PGT151 does not affect the orientation of 10E8 directly by steric blocking, we assume the differing orientation of Fab C is rather mediated by the stabilization or asymmetric distortion of the

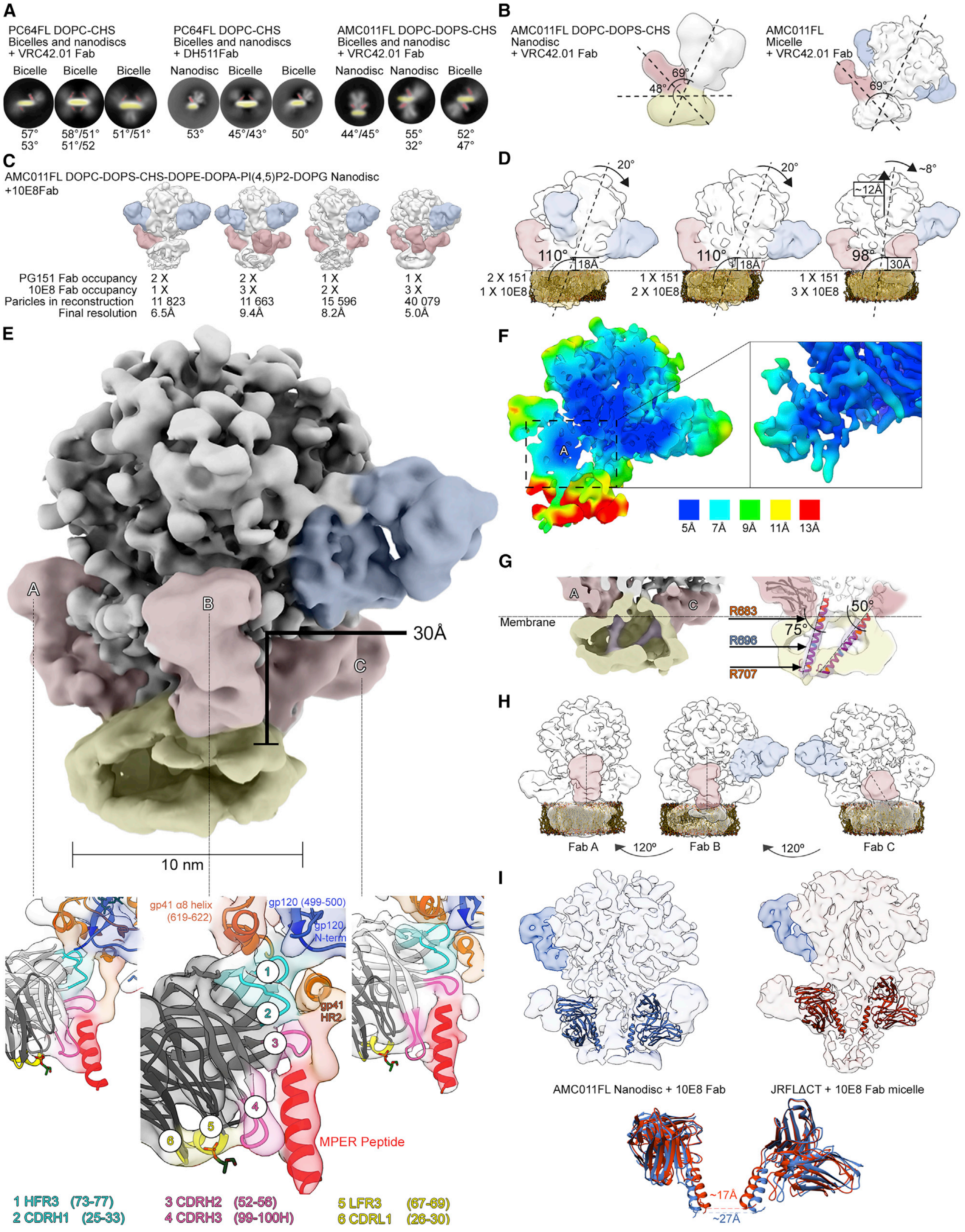

(legend on next page)

protomer-protomer interface induced by PGT151. When fitted positions of 10E8 Fab-MPER peptide crystal structures in the AMC011 nanodisc were compared to ones fitted in the JRFLΔCT micelle complex (Lee et al., 2016), the membrane-surface-facing sides of the MPER peptides were ~10Å closer to each other (Figure 4I).

Assembly of Engineered Env in Peptidiscs

To overcome the requirement of using antibodies as a purification and stabilization reagent and to adapt our methods for immunogen design purposes, we introduced stabilizing mutations to Env. BG505 Env was engineered with additional mutations (termed SOSIPv5.2) (Torrents de la Peña et al., 2017) and MD39 mutations (Steichen et al., 2016) that have previously been demonstrated to increase the stability and expression of soluble trimeric ectodomain of BG505. In addition, the construct was truncated at the C terminus at residue 710 to eliminate unwanted epitopes. This construct was named BG505-ST-710. A soluble version of this construct was generated by further MPER and TMD deletion and named BG505-ST-664. The introduced modifications allowed purification workflows for BG505-ST-710 similar to soluble SOSIP versions and production of unliganded TMD containing trimer. To reduce the introduction of potentially immunogenically active elements from the scaffold protein and improve assembly efficiency, we utilized an apolipoprotein-A1-derived bi-helical peptidisc scaffold (Carlson et al., 2018). Assembly with the peptidisc scaffold was also reproducibly more efficient with similar or higher Env occupancy per disc than with MSP1D1. Peptidisc showed similar MPER binding modes as PC64FL and AMC011FL nanodiscs, apart from MPER Fab complex classes, where Env was tilted over the side of the disc, most likely because of the lack of anchoring by CTD, a less restricted scaffold belt formed by the peptidisc scaffold, or the combined effect of both (Figures 5A and S6). Consequently, this appeared also to allow binding of an additional MPER Fab on the other side of the disc plane as seen in 2D class averages of complexes with DH511.2, PGZL1, and VRC42.01 Fabs (Figure S6). Binding of this construct to a panel of antibodies was tested by biolayer interferometry (BLI; Octet) using lectin-based Env capture (Figure 5B). Quaternary-specific Fab PGT145, which preferentially binds to correctly folded trimers, bound to Env peptidiscs with binding levels (Figure 5B) similar to soluble BG505-ST-664. Binding levels of PGT151 to Env peptidiscs were lower compared to the soluble trimer (Figure 5B). Non-neutralizing antibody B6, which binds only to non-native forms of Env, and 39F, which recognizes non-neutralizing epitopes in the V3 loop, showed minimal reactivity to both samples (Figure 5B). 10E8 bound to Env peptidiscs, but not to BG505-ST-664, which lacks the MPER epitope (Figure 5B). Taken together, these data confirm that engineered Env peptidiscs are predominantly in native pre-fusion conformations similar to soluble BG505-ST-664 and have the capacity to bind MPER antibodies. To further validate the assembly platform, we compared the antigenicity to AMC011FL in detergent-lipid micelle and nanodisc with DOPC-DOPS-CHS lipid composition. As shown earlier with micelle-embedded AMC011FL, we observed weak binding to VRC01 antibody and clear binding to the quaternary-specific apex-targeting bNAb PGT145 (Torrents de la Peña et al., 2019), indicating that at least a subpopulation of trimers are in a native conformation (Figure 5C). Binding of non-neutralizing monoclonal antibodies (mAbs) 39F (V3-loop) and B6 (CD4 binding site) was very strong, as expected for wild-type Env proteins in the absence of stabilizing mutations and also in agreement with EM analysis of a similarly prepared nanodisc, where raw data and 2D and 3D class averages showed dissociating trimers and heterogenous Env incorporation into the bilayer (Figures S3, S4, and S6). MPER bNAb 10E8 bound much weaker to the nanodisc than to the micelle, consistent with a more restricted angle of approach imposed by lipid membrane in nanodiscs, whereas membrane-interaction-optimized 10E8v4-5R+100cF showed markedly higher binding to nanodisc as compared to micelle (Figure 5C).

DISCUSSION

Here, we developed lipid assembly methods and demonstrate that these, in combination with single-particle EM analysis, are well suited to improve our understanding of the structural

Figure 4. Analysis of FL Env in Lipid Bicelles and Nanodiscs in Complex with MPER-Targeting Fabs

(A) Representative 2D class averages from negative-stain EM data from lipid assemblies of PC64FL and AMC011FL in complex with MPER-targeting antibodies. In all bilayer-assembled Env-MPER antibody complexes, Env was tilted at varying degrees and displaced to the edge of the bilayer. The degree of the Fab binding angle is estimated in relation to the bilayer from the given 2D class average. The lipid bilayer is highlighted in yellow, and MPER Fab is shown in red.

(B) Comparison of the VRC42.01 Fab-binding angle in the nanodisc and micelle. The angle between the Fab and the ectodomain was identical, while in the nanodisc, an additional angle can be measured between the bilayer and the Fab. PGT151 Fab is highlighted in blue throughout the figure.

(C) Low-pass filtered cryo-EM reconstructions of the AMC011FL nanodisc in complex with 10E8 Fab with different Fab occupancies. The highest resolution and particle count were obtained with the complex containing one copy of PGT151 Fab and three copies of 10E8 Fab, which is used for (E)–(I).

(D) Low-pass-filtered reconstructions with one, two, or three copies of 10E8 Fab showing degree of tilting and distance of Env from bilayer in different 10E8 Fab occupancy states.

(E) Highest resolution reconstruction and epitopes of the three Fabs with 10E8 Fab crystal structure (PDB: 5T80) docked in. Epitope components are highlighted as indicated in the panel below. The distance from the bilayer surface is also indicated.

(F) Local resolution estimation showing up to 5 Å resolution in the ectodomain and stabilized 10E8 Fab epitope.

(G) Fab A and Fab C stabilized TMD orientations are highlighted in purple. Residues marking the outer (R683) and inner (R707) bilayer surfaces are highlighted in orange and R696 in blue, which marks the crossing point of TMDs in micelle samples and is now separated. The angles of the two TMDs are estimated in relation to the bilayer surface.

(H) Two Fab orientations and dependency of Fab C on the PGT151 position are highlighted in red in low-pass-filtered maps.

(I) Comparison of 10E8 Fab docking into the AMC011FL nanodisc and JRFLΔCT micelle reconstructions (EMD-3312) showing an ~10 Å change in the distance between the MPER peptides (residue Q135 in PDB: 5T80).

See also Figures S2, S4, and S6 and Table S1.

CellPress
OPEN ACCESS

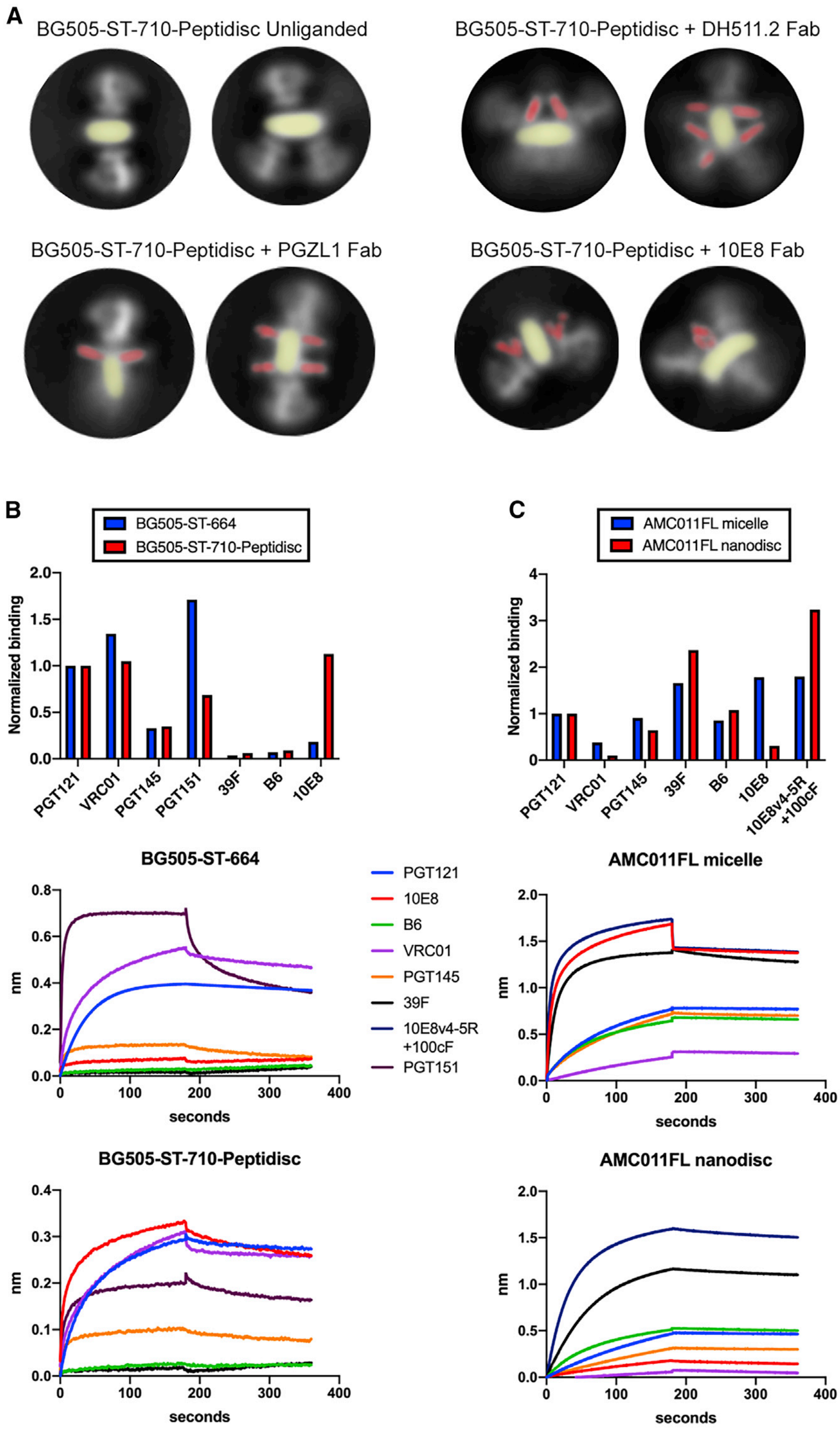

Figure 5. Assembly of Vaccine Design Adapted BG505-ST-710 into Peptidiscs

(A) Examples of BG505-ST-710 peptidiscs with and without the addition of MPER-targeting Fabs as determined from negative-stain EM 2D class averages.

(B) Antigenic profiling of BG505-ST-710 peptidiscs compared to equivalent soluble Env (BG505-ST-664) by lectin capture-based biolayer interferometry. The top graph shows binding signals of the indicated Fabs after normalization to the respective PGT121 signal. Exemplary traces of the indicated Fabs are shown below.

(C) Antigenicity comparison of AMC011FL in detergent micelles and lipid nanodiscs with protein A-IgG capture mode.

See also Table S1.

dynamics of the HIV-1 Env glycoprotein. The system is currently limited to either PGT151-stabilized FL, wild-type constructs or constructs with stabilizing mutations in the ectodomain. In addition, the assemblies are small patches of membrane that may limit the mobility and flexibility of the Env trimer. Nevertheless, the system is a step toward more native environment and open new possibilities for studying Env structure in lipid bilayers by single-particle EM analysis. In addition to controlled lipid composition and the testing new MPER antibodies such as 10E8v4-5R+100cF with improved lipid contacts (Kwon et al., 2018b), these membrane-embedded Envs may allow structural studies of the effect that CD4 receptor engagement has on the neutralization mechanism of the MPER bNAbs, which thus far has not been possible. As these complexes are likely heterogenous, excellent scalability and the classification power of single-particle EM analysis becomes an important factor in addition to being able to yield high-resolution structural data. Systematic studies of lipid composition, larger macromolecular complexes, and immunization trials will therefore be subjects of future research.

Our studies demonstrate that the ectodomains of membrane-embedded Envs are stable when bound to PGT151 or stabilized with mutations, while the MPER and TMD exhibit a large degree of positional and local structural heterogeneity reflective of local dynamics near the membrane. Large dynamic shifts have also been observed with single-molecule Förster resonance energy transfer (smFRET) studies that suggest at least three distinct conformational states (Lu et al., 2019; Munro et al., 2014). In the light of these studies, the flexibility and dynamics we observe in the lipid assemblies may reflect similar spontaneous sampling of functional Env conformations. Recent advances in cryoelectron tomography methods offer another promising approach to study the structures of Env exposed on the virus particle surface. Thus far, these studies have not reached higher than ~20 Å resolution (Liu et al., 2008; Meyerson et al., 2013) and may be limited by the conformational sampling observed with

Table 1. Contacting Regions between Env and MPER-Targeting Antibody 10E8 Mapped from the Cryo-EM Reconstruction of AMC011FL Nanodisc in Complex with the Fab

Env	10E8 Fab
Ectodomain	
gp41 α8-helix (residues 619-622)	CDRH1 (25-33)
gp120 residues (499-500)	HC FR3 (73-77)
gp120 N terminus	
Ectodomain-MPER	
HR2	CDRH2 (52-56)
MPER peptide	CDRH3 (99-100H)
Lipids/bilayer	CDRL1 (26-30)
	LC FR3 (67-69)

smFRET measurements and with our lipid assembly system (Figure 2E). In the lipid systems presented here, structural heterogeneity could be reduced by the addition of MPER Fabs or by incorporation into a nanodisc. Although we did not achieve high enough resolution to build atomic models for MPER, TMD, or CTD, we could extract new details of these typically dynamic domains through 3D classification and by docking high-resolution structures of complex components into the reconstructions.

For the PC64FL micelle in complex with VRC42.01 Fab and the AMC011FL nanodisc with 10E8 Fab, densities likely corresponding to the MPER-bound TMD could be traced through the micelle and nanodisc, respectively. Interestingly, a tilted orientation of TMDs could be determined in micelles with a crossing point at conserved residue R696. Influenza hemagglutinin was shown to have similar TMD dynamics with tilted and straight orientations, suggesting that these could be common type I viral fusion protein TMD topologies (Benton et al., 2018). The arrangement of helices shown here for HIV Env is different than the three-helix bundle topology observed in NMR structures of the TM helices alone (Chen and Chou, 2017; Chiliveri et al., 2018; Dev et al., 2016; Hollingsworth et al., 2018; Kwon et al., 2018a). Thus, the compact three-helix bundle conformation likely represents the low-energy postfusion conformation of the TMD, and its formation is preferred in the minimal constructs used in the NMR and MD studies. More recently, a similar study of isolated MPER-TMD peptide in nanodiscs supported our conclusions that, in a more native lipid environment, a stable, trimeric topology of the TMD is unfavorable (Wang et al., 2019). Our data in detergent-lipid micelles demonstrate how the ectodomain and MPER restrain the TMD in a crossed topology, which is particularly apparent in the MPER Fab-bound state. This topology of the TMD is consistent with the meta-stable prefusion state primed for the energetically downhill conformational changes associated with post-fusion conformation. In the nanodisc environment, an additional 25° more vertical TMD orientation was observed (Figure 4G). Strikingly, a similar long, straight MPER-TMD helix was recently shown in a crystal structure of LN01 antibody in complex with complete TMD (Pinto et al., 2019). In the nanodisc structure presented here, the R696 TMD crossing point seen in the micelle (Figure 3B) is now disconnected from other protomers, suggesting that TMD domain coordination is influenced by MPER antibody binding. As compared to the micelle, the nanodisc bilayer introduces additional support for Fab binding, which may result in a more native environment and allow for separation of TMDs in contrast to micelle-embedded complex. The inference is also supported by the difference in position of 10E8 Fab in the AMC011FL nanodisc as compared to the JRFLΔCT micelle, where the membrane-surface-facing side of the docked Fab-MPER peptide structure (PDB: 5T80) is ~10 Å closer to the adjacent protomer as compared to the nanodisc (Figure 4I). Therefore, the micelle appears to allow closer juxtaposition of MPER peptides from adjacent protomers when bound to the Fab. Alternatively, the difference in MPER Fab-bound protomer TMD topology could be due to differential effects imposed by the VRC42.01 and 10E8 antibodies.

Our data demonstrate that the MPER is sterically difficult to access and thus support a progressive binding model for MPER antibodies that occurs in series of steps (Figure 6). Initial contacts of antibody between the Env ectodomain and the lipid surface lead to tilting of Env, which may occur through gradual stabilization of the sampled conformational states. This interaction in turn increases the exposure of the MPER peptide by partially lifting it off from the membrane surface (Figure 4), lending support that the membrane-embedded and exposed or lifted conformation of the MPER peptide are both relevant for binding (Fu et al., 2018; Irimia et al., 2017; Kwon et al., 2018a). Binding of additional MPER antibodies may temporarily destabilize the ectodomain and in this way contribute to increased shedding of gp120 shown in an earlier study (Ruprecht et al., 2011). This model would also be in agreement with studies showing increased neutralization efficiency after binding of the CD4 receptor (Kim et al., 2014; Rathinakumar et al., 2012), which results in a steeper angle of HR2, the helix immediately N-terminal to the MPER (Ozorowski et al., 2017). In the context of the Env clustering on the mature virus particle surface and formation of the entry claw (Carravilla et al., 2019; Sougrat et al., 2007), the tilting of two Envs away from each other would also inhibit the formation of the entry claw and possibly contribute to the neutralization efficiency. The tilting component could be mechanistically related to the binding of 35O22, another bNAb that targets an epitope at the interface of gp41 and gp120 and similarly binds better after CD4 receptor engagement (Huang et al., 2014). Finally, given the geometry of the three MPERs in trimeric Env, the two Fab arms of an intact immunoglobulin G (IgG) would preferentially bind to two different Env trimers rather than to a single Env spike as seen on the PC64FL bicelle surface with DH511.2 IgG (Figure S6). Given the limitations of the lipid assembly system, the stoichiometry of MPER antibody arms may, however, be different in the biological context where membrane surface is circular and Env is more prone to shed gp120. Second and third antibody arm binding to same trimer on the virus particle may therefore encounter an Env that has already shed gp120 and does not need tilting to access the MPER.

The structures with 10E8 yield insight into its mode of binding and possible developmental pathways. As observed in the complex of JRFLΔCT with 10E8 (Lee et al., 2016), our data illustrate that glycans N88 and N625 are positioned to clash with the antibody. These clashes would occur in the proximity of FR3 of the 10E8 HC. FR3 likely also has peptide contacts with gp120 and gp41 (Table 1) and is a component of 10E8 paratope on the lipid surface and ectodomain. In another study, 10E8 still showed

CellPress
OPEN ACCESS

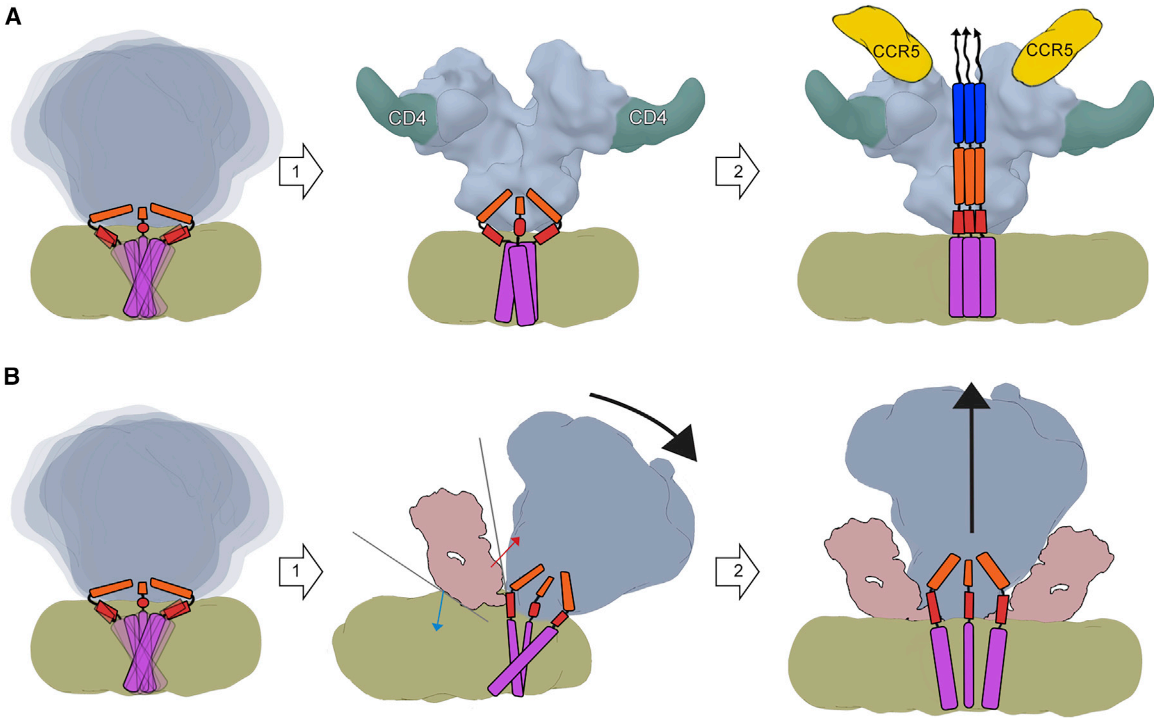

Figure 6. Model for MPER-Targeting Neutralization Mechanism Steps Based on Lipid Bilayer Assemblies

Suggested HR1 (blue), HR2 (orange), MPER (red), and TMD (purple) orientations are presented. Based on the lack of density corresponding to the TMD in our EM reconstructions, we hypothesize that in their ground state, TMDs are fluctuating in a loosely folded scissoring motion. Similarly, native Env spontaneously samples different functional states.

(A) In the absence of an MPER-targeting Fab, (1) CD4 triggers stabilization and conformational changes in HR2, MPER and TMD in addition to opening of the ectodomain and exposure of the CCR5 coreceptor binding site. (2) This leads to coreceptor binding and formation of a lower energy state post-fusion conformation of fusion peptide (arrows), HR1, HR2, MPER, and TMD. Further downstream (not represented in this schematic), this extended three-helix bundle undergoes a further condensation into a six-helix bundle and, in concert with adjacent Env molecules, facilitates membrane fusion and viral entry into the host cell.

(B) In the MPER-targeting neutralization path (1), approaching antibody forms a wedge between the ectodomain (red arrow) and bilayer surface (blue arrow), tilting the ectodomain, increasing the exposure of MPER peptide, and stabilizing the scissoring of MPER-TMD. While on a planar bilayer, the tilting may restrict access to additional MPER epitopes within the trimer, we observe at least a second MPER-binding event in all tested assemblies. (2) Thus, subsequent Fab arms binding to other protomers may eventually lead to displacement or increased shedding of gp120 in the absence of stabilizing mutations or PGT151 Fab. MPER-TMDs are now separated and locked by the MPER antibody.

substantial, albeit reduced, neutralization activity when the FRs were reverted closer to the germline sequence (Georgiev et al., 2014). Importantly, however, these germline revertants had mature CDRs grafted into the constructs and, therefore, the significance of the FRs to early antibody maturation events may have been lost. It is also important to note that framework mutations are shown to be generally required for broad neutralization (Klein et al., 2013). Thus, in light of these studies, our results suggest that immunogens that can drive mutations in FR3 may be important in triggering the maturation of MPER bNAbs.

Most MPER immunogen design efforts have focused on using the MPER peptide in isolation (Liu et al., 2018), largely because of the difficulty in producing FL Env. The critical challenges in using transmembrane Env versions are the low protein production level and instability of the purified transmembrane protein. We achieved ~100–300 μg total yield per 1 L 293F cells for stabilized BG505-ST-710, which is roughly an order of magnitude away from what would be required for immunization trials in large animal models. Furthermore, compositional heterogeneity could complicate immunogen formulation, although higher Env occupancy per trimer may also be a beneficial factor in immunogen display. With BG505-ST-710, we observed slightly higher compositional heterogeneity as compared to FL Env nanodiscs with one to six Envs per assembly. The combined effect of shorter bi-helical scaffold and the lack of CTD may allow more Envs to be incorporated into single assembly. Likely due to same reasons, upon MPER Fab binding, Env in the peptidisc was displaced over the side of the disc. Nonetheless, with the BG505-ST-710 peptidisc, we were able to display full MPER epitopes in a single, stable molecule. Importantly, we observed the contacts to α8-helix of gp41 in both micelle and nanodisc samples (Figures 4 and S5), indicating that this may be a conserved contact point between MPER antibodies and the ectodomain and a contributor in the tilting component of the neutralization mechanism. The exact structural details should therefore be studied with point mutations in combination with other stabilizing mutations to explore the significance of this contact for antibody binding. In the engineered BG505-ST-710 construct, stabilizing mutations allowed the purification protocol to closely follow

the methods standardized for soluble Env, enabling similar structure-based immunogen design approaches as for soluble constructs. Env-nanodisc immunization could address three important open questions in HIV vaccine design. First, the transmembrane version of Env has been shown to possess a glycan shield that is closer to the composition of the glycan shield presented on virus particles (Cao et al., 2018; Rantalainen et al., 2018; Seabright et al., 2019; Torrents de la Peña et al., 2019). Second, a recent analysis of the polyclonal immune response against soluble SOSIP immunogen indicated that in the non-human primate model, a dominant response targeted the exposed base of the trimer (Bianchi et al., 2018). In membrane-embedded formulations, this immunodominant neoepitope would be protected by the bilayer. Additional prevention of off-target immune responses against the scaffold protein can also be minimized with less immunoreactive peptide scaffolds, such as A22 (Kuai et al., 2017, 2018) and peptidisc (Carlson et al., 2018). The third important factor in Env-nanodisc immunization would be the capacity to present the full set of quaternary epitopes, which more closely resemble the corresponding epitopes presented on native virus particles. In summary, our structures enabled examination of the full MPER epitope in a quaternary context (Figure 4E; Table 1). These advances will enable future studies of Env in its native, membrane-embedded environment, contributing to immunogen design for an effective HIV vaccine.

STAR★METHODS

Detailed methods are provided in the online version of this paper and include the following:

- KEY RESOURCES TABLE
- RESOURCE AVAILABILITY
  - Lead Contact
  - Materials Availability
  - Data and Code Availability
- EXPERIMENTAL MODEL AND SUBJECT DETAILS
- METHOD DETAILS
  - Recombinant protein expression and purification
  - IgG and Fab expression and purification
  - Lipid stock preparation
  - Assembly
  - Mass spectrometric identification of lipids
  - Bio-layer interferometry
  - Electron microscopy sample preparation
  - Electron microscopy imaging and data processing
- QUANTIFICATION AND STATISTICAL ANALYSIS

SUPPLEMENTAL INFORMATION

Supplemental Information can be found online at https://doi.org/10.1016/j.celrep.2020.107583.

ACKNOWLEDGMENTS

The authors would like to thank Hannah Turner and Bill Anderson for assistance with microscope management and operation, Charles Bowman and J.C. Ducom for assistance with EM data management, and Lauren Holden for critical reading of the manuscript. DH511.2 Fab and IgG were generous gifts from Barton F. Haynes (Duke University School of Medicine, Durham, NC). This work was supported by the NIH Intramural Research Program of the Vaccine Research Center, National Institute of Allergy and Infectious Diseases (grants UM1AI100663 [I.A.W., W.R.S., and A.B.W.], UM1 AI144462 [I.A.W., W.R.S., and A.B.W.], and R01 AI143563 [M.B.Z.]) and the Bill and Melinda Gates Foundation through the Collaboration for AIDS Vaccine Discovery (CAVD) grant OPP1115782 (A.B.W.). This is manuscript number 29908 from Scripps Research.

AUTHOR CONTRIBUTIONS

K.R. designed the research and adapted the lipid assembly methods for Env. K.R., A.A., W.-H.S., and J.L.T. prepared Env samples. K.R. prepared EM samples. K.R., W.-H.S., and Z.B. imaged EM data. K.R. and Z.B. processed EM data. A.A. and W.-H.S. engineered Env constructs. A.A. and K.R. adapted lipid assembly method to immunogen production. T.S. performed BLI experiments. X.Z. performed mass spectrometry (MS) analyses. L.Z., A.I., J.C., K.Z., Y.D.K., W.L., C.A.S., and R.V. provided purified IgG and Fabs. K.R., T.S., and X.Z. prepared the figures. K.R. and A.B.W. wrote the manuscript. S.K., P.D.K., N.A.D.-R., I.A.W., M.B.Z., J.R.Y. III, W.R.S., and A.B.W. provided the resources and supervised the project.

DECLARATION OF INTERESTS

The authors declare no competing interests.

Received: November 14, 2019
Revised: March 5, 2020
Accepted: April 7, 2020
Published: April 28, 2020

REFERENCES

Bayburt, T.H., Grinkova, Y.V., and Sligar, S.G. (2002). Self-assembly of discoidal phospholipid bilayer nanoparticles with membrane scaffold proteins. Nano Lett. *2*, 853–856.

Benton, D.J., Nans, A., Calder, L.J., Turner, J., Neu, U., Lin, Y.P., Ketelaars, E., Kallewaard, N.L., Corti, D., Lanzavecchia, A., et al. (2018). Influenza hemagglutinin membrane anchor. Proc. Natl. Acad. Sci. USA *115*, 10112–10117.

Bianchi, M., Turner, H.L., Nogal, B., Cottrell, C.A., Oyen, D., Pauthner, M., Bastidas, R., Nedellec, R., McCoy, L.E., Wilson, I.A., et al. (2018). Electron-microscopy-based epitope mapping defines specificities of polyclonal antibodies elicited during HIV-1 BG505 envelope trimer immunization. Immunity *49*, 288–300.e288.

Blattner, C., Lee, J.H., Sliepen, K., Derking, R., Falkowska, E., de la Peña, A.T., Cupo, A., Julien, J.P., van Gils, M., Lee, P.S., et al. (2014). Structural delineation of a quaternary, cleavage-dependent epitope at the gp41-gp120 interface on intact HIV-1 Env trimers. Immunity *40*, 669–680.

Cao, L., Pauthner, M., Andrabi, R., Rantalainen, K., Berndsen, Z., Diedrich, J.K., Menis, S., Sok, D., Bastidas, R., Park, S.R., et al. (2018). Differential processing of HIV envelope glycans on the virus and soluble recombinant trimer. Nat. Commun. *9*, 3693.

Cardoso, R.M., Zwick, M.B., Stanfield, R.L., Kunert, R., Binley, J.M., Katinger, H., Burton, D.R., and Wilson, I.A. (2005). Broadly neutralizing anti-HIV antibody 4E10 recognizes a helical conformation of a highly conserved fusion-associated motif in gp41. Immunity *22*, 163–173.

Carlson, M.L., Young, J.W., Zhao, Z., Fabre, L., Jun, D., Li, J., Li, J., Dhupar, H.S., Wason, I., Mills, A.T., et al. (2018). The Peptidisc, a simple method for stabilizing membrane proteins in detergent-free solution. eLife *7*, e34085.

Carravilla, P., Chojnacki, J., Rujas, E., Insausti, S., Largo, E., Waithe, D., Apellaniz, B., Sicard, T., Julien, J.P., Eggeling, C., and Nieva, J.L. (2019). Molecular recognition of the native HIV-1 MPER revealed by STED microscopy of single virions. Nat. Commun. *10*, 78.

Chen, B. (2019). Molecular mechanism of HIV-1 entry. Trends Microbiol. *27*, 878–891.

Chen, B., and Chou, J.J. (2017). Structure of the transmembrane domain of HIV-1 envelope glycoprotein. FEBS J. *284*, 1171–1177.

Chiliveri, S.C., Louis, J.M., Ghirlando, R., Baber, J.L., and Bax, A. (2018). Tilted, uninterrupted, monomeric HIV-1 gp41 transmembrane helix from residual dipolar couplings. J. Am. Chem. Soc. *140*, 34–37.

Cooper, R.S., Georgieva, E.R., Borbat, P.P., Freed, J.H., and Heldwein, E.E. (2018). Structural basis for membrane anchoring and fusion regulation of the herpes simplex virus fusogen gB. Nat. Struct. Mol. Biol. *25*, 416–424.

Denisov, I.G., and Sligar, S.G. (2016). Nanodiscs for structural and functional studies of membrane proteins. Nat. Struct. Mol. Biol. *23*, 481–486.

Denisov, I.G., Grinkova, Y.V., Lazarides, A.A., and Sligar, S.G. (2004). Directed self-assembly of monodisperse phospholipid bilayer Nanodiscs with controlled size. J. Am. Chem. Soc. *126*, 3477–3487.

Dev, J., Park, D., Fu, Q., Chen, J., Ha, H.J., Ghantous, F., Herrmann, T., Chang, W., Liu, Z., Frey, G., et al. (2016). Structural basis for membrane anchoring of HIV-1 envelope spike. Science *353*, 172–175.

Dey, A.K., Cupo, A., Ozorowski, G., Sharma, V.K., Behrens, A.J., Go, E.P., Ketas, T.J., Yasmeen, A., Klasse, P.J., Sayeed, E., et al. (2018). cGMP production and analysis of BG505 SOSIP.664, an extensively glycosylated, trimeric HIV-1 envelope glycoprotein vaccine candidate. Biotechnol. Bioeng. *115*, 885–899.

Efremov, R.G., Gatsogiannis, C., and Raunser, S. (2017). Lipid nanodiscs as a tool for high-resolution structure determination of membrane proteins by single-particle cryo-EM. Methods Enzymol. *594*, 1–30.

Fu, Q., Shaik, M.M., Cai, Y., Ghantous, F., Piai, A., Peng, H., Rits-Volloch, S., Liu, Z., Harrison, S.C., Seaman, M.S., et al. (2018). Structure of the membrane proximal external region of HIV-1 envelope glycoprotein. Proc. Natl. Acad. Sci. USA *115*, E8892–E8899.

Georgiev, I.S., Rudicell, R.S., Saunders, K.O., Shi, W., Kirys, T., McKee, K., O'Dell, S., Chuang, G.Y., Yang, Z.Y., Ofek, G., et al. (2014). Antibodies VRC01 and 10E8 neutralize HIV-1 with high breadth and potency even with Ig-framework regions substantially reverted to germline. J. Immunol. *192*, 1100–1106.

Goddard, T.D., Huang, C.C., Meng, E.C., Pettersen, E.F., Couch, G.S., Morris, J.H., and Ferrin, T.E. (2018). UCSF ChimeraX: Meeting modern challenges in visualization and analysis. Protein Sci. *27*, 14–25.

Guenaga, J., Garces, F., de Val, N., Stanfield, R.L., Dubrovskaya, V., Higgins, B., Carrette, B., Ward, A.B., Wilson, I.A., and Wyatt, R.T. (2017). Glycine substitution at helix-to-coil transitions facilitates the structural determination of a stabilized subtype C HIV envelope glycoprotein. Immunity *46*, 792–803.e3.

Hammonds, J., Chen, X., Ding, L., Fouts, T., De Vico, A., zur Megede, J., Barnett, S., and Spearman, P. (2003). Gp120 stability on HIV-1 virions and Gag-Env pseudovirions is enhanced by an uncleaved Gag core. Virology *314*, 636–649.

Harrison, S.C. (2015). Viral membrane fusion. Virology *479-480*, 498–507.

Haynes, B.F., and Mascola, J.R. (2017). The quest for an antibody-based HIV vaccine. Immunol. Rev. *275*, 5–10.

Hollingsworth, L.R., 4th, Lemkul, J.A., Bevan, D.R., and Brown, A.M. (2018). HIV-1 Env gp41 transmembrane domain dynamics are modulated by lipid, water, and ion interactions. Biophys. J. *115*, 84–94.

Huang, J., Ofek, G., Laub, L., Louder, M.K., Doria-Rose, N.A., Longo, N.S., Imamichi, H., Bailer, R.T., Chakrabarti, B., Sharma, S.K., et al. (2012). Broad and potent neutralization of HIV-1 by a gp41-specific human antibody. Nature *491*, 406–412.

Huang, J., Kang, B.H., Pancera, M., Lee, J.H., Tong, T., Feng, Y., Imamichi, H., Georgiev, I.S., Chuang, G.Y., Druz, A., et al. (2014). Broad and potent HIV-1 neutralization by a human antibody that binds the gp41-gp120 interface. Nature *515*, 138–142.

Irimia, A., Sarkar, A., Stanfield, R.L., and Wilson, I.A. (2016). Crystallographic identification of lipid as an integral component of the epitope of HIV broadly neutralizing antibody 4E10. Immunity *44*, 21–31.

Irimia, A., Serra, A.M., Sarkar, A., Jacak, R., Kalyuzhniy, O., Sok, D., Saye-Francisco, K.L., Schiffner, T., Tingle, R., Kubitz, M., et al. (2017). Lipid interactions and angle of approach to the HIV-1 viral membrane of broadly neutralizing antibody 10E8: Insights for vaccine and therapeutic design. PLoS Pathog. *13*, e1006212.

Jo, S., Kim, T., Iyer, V.G., and Im, W. (2008). CHARMM-GUI: a web-based graphical user interface for CHARMM. J. Comput. Chem. *29*, 1859–1865.

Joyce, M.G., Georgiev, I.S., Yang, Y., Druz, A., Geng, H., Chuang, G.Y., Kwon, Y.D., Pancera, M., Rawi, R., Sastry, M., et al. (2017). Soluble prefusion closed DS-SOSIP.664-Env trimers of diverse HIV-1 strains. Cell Rep. *21*, 2992–3002.

Kim, A.S., Leaman, D.P., and Zwick, M.B. (2014). Antibody to gp41 MPER alters functional properties of HIV-1 Env without complete neutralization. PLoS Pathog. *10*, e1004271.

Klein, F., Diskin, R., Scheid, J.F., Gaebler, C., Mouquet, H., Georgiev, I.S., Pancera, M., Zhou, T., Incesu, R.B., Fu, B.Z., et al. (2013). Somatic mutations of the immunoglobulin framework are generally required for broad and potent HIV-1 neutralization. Cell *153*, 126–138.

Krebs, S.J., Kwon, Y.D., Schramm, C.A., Law, W.H., Donofrio, G., Zhou, K.H., Gift, S., Dussupt, V., Georgiev, I.S., Schatzle, S., et al. (2019). Longitudinal analysis reveals early development of three MPER-directed neutralizing antibody lineages from an HIV-1-infected individual. Immunity *50*, 677–691.e613.

Kuai, R., Ochyl, L.J., Bahjat, K.S., Schwendeman, A., and Moon, J.J. (2017). Designer vaccine nanodiscs for personalized cancer immunotherapy. Nat. Mater. *16*, 489–496.

Kuai, R., Sun, X., Yuan, W., Xu, Y., Schwendeman, A., and Moon, J.J. (2018). Subcutaneous nanodisc vaccination with neoantigens for combination cancer immunotherapy. Bioconjug. Chem. *29*, 771–775.

Kulp, D.W., Steichen, J.M., Pauthner, M., Hu, X., Schiffner, T., Liguori, A., Cottrell, C.A., Havenar-Daughton, C., Ozorowski, G., Georgeson, E., et al. (2017). Structure-based design of native-like HIV-1 envelope trimers to silence non-neutralizing epitopes and eliminate CD4 binding. Nat. Commun. *8*, 1655.

Kwon, Y.D., Pancera, M., Acharya, P., Georgiev, I.S., Crooks, E.T., Gorman, J., Joyce, M.G., Guttman, M., Ma, X., Narpala, S., et al. (2015). Crystal structure, conformational fixation and entry-related interactions of mature ligand-free HIV-1 Env. Nat. Struct. Mol. Biol. *22*, 522–531.

Kwon, B., Lee, M., Waring, A.J., and Hong, M. (2018a). Oligomeric structure and three-dimensional fold of the HIV gp41 membrane-proximal external region and transmembrane domain in phospholipid bilayers. J. Am. Chem. Soc. *140*, 8246–8259.

Kwon, Y.D., Chuang, G.Y., Zhang, B., Bailer, R.T., Doria-Rose, N.A., Gindin, T.S., Lin, B., Louder, M.K., McKee, K., O'Dell, S., et al. (2018b). Surface-matrix screening identifies semi-specific interactions that improve potency of a near pan-reactive HIV-1-neutralizing antibody. Cell Rep. *22*, 1798–1809.

Landais, E., Murrell, B., Briney, B., Murrell, S., Rantalainen, K., Berndsen, Z.T., Ramos, A., Wickramasinghe, L., Smith, M.L., Eren, K., et al.; IAVI Protocol C Investigators; IAVI African HIV Research Network (2017). HIV Envelope Glycoform Heterogeneity and Localized Diversity Govern the Initiation and Maturation of a V2 Apex Broadly Neutralizing Antibody Lineage. Immunity *47*, 990-1003.e9.

Lee, J.H., Ozorowski, G., and Ward, A.B. (2016). Cryo-EM structure of a native, fully glycosylated, cleaved HIV-1 envelope trimer. Science *351*, 1043–1048.

Liu, J., Bartesaghi, A., Borgnia, M.J., Sapiro, G., and Subramaniam, S. (2008). Molecular architecture of native HIV-1 gp120 trimers. Nature *455*, 109–113.

Liu, H., Su, X., Si, L., Lu, L., and Jiang, S. (2018). The development of HIV vaccines targeting gp41 membrane-proximal external region (MPER): challenges and prospects. Protein Cell *9*, 596–615.

Lorizate, M., Sachsenheimer, T., Glass, B., Habermann, A., Gerl, M.J., Kräusslich, H.G., and Brügger, B. (2013). Comparative lipidomics analysis of HIV-1 particles and their producer cell membrane in different cell lines. Cell. Microbiol. *15*, 292–304.

Lu, M., Ma, X., Castillo-Menendez, L.R., Gorman, J., Alsahafi, N., Ermel, U., Terry, D.S., Chambers, M., Peng, D., Zhang, B., et al. (2019). Associating HIV-1 envelope glycoprotein structures with states on the virus observed by smFRET. Nature *568*, 415–419.

Meyerson, J.R., Tran, E.E., Kuybeda, O., Chen, W., Dimitrov, D.S., Gorlani, A., Verrips, T., Lifson, J.D., and Subramaniam, S. (2013). Molecular structures of trimeric HIV-1 Env in complex with small antibody derivatives. Proc. Natl. Acad. Sci. USA *110*, 513–518.

Munro, J.B., Gorman, J., Ma, X., Zhou, Z., Arthos, J., Burton, D.R., Koff, W.C., Courter, J.R., Smith, A.B., 3rd, Kwong, P.D., et al. (2014). Conformational dynamics of single HIV-1 envelope trimers on the surface of native virions. Science *346*, 759–763.

Nakane, T., Kimanius, D., Lindahl, E., and Scheres, S.H. (2018). Characterisation of molecular motions in cryo-EM single-particle data by multi-body refinement in RELION. eLife *7*, e36861.

Ozorowski, G., Pallesen, J., de Val, N., Lyumkis, D., Cottrell, C.A., Torres, J.L., Copps, J., Stanfield, R.L., Cupo, A., Pugach, P., et al. (2017). Open and closed structures reveal allostery and pliability in the HIV-1 envelope spike. Nature *547*, 360–363.

Pettersen, E.F., Goddard, T.D., Huang, C.C., Couch, G.S., Greenblatt, D.M., Meng, E.C., and Ferrin, T.E. (2004). UCSF Chimera–a visualization system for exploratory research and analysis. J. Comput. Chem. *25*, 1605–1612.

Pinto, D., Fenwick, C., Caillat, C., Silacci, C., Guseva, S., Dehez, F., Chipot, C., Barbieri, S., Minola, A., Jarrossay, D., et al. (2019). Structural basis for broad HIV-1 neutralization by the MPER-specific human broadly neutralizing antibody LN01. Cell Host Microbe. *26*, 623–637.e8.

Potter, C.S., Chu, H., Frey, B., Green, C., Kisseberth, N., Madden, T.J., Miller, K.L., Nahrstedt, K., Pulokas, J., Reilein, A., et al. (1999). Leginon: a system for fully automated acquisition of 1000 electron micrographs a day. Ultramicroscopy *77*, 153–161.

Punjani, A., Rubinstein, J.L., Fleet, D.J., and Brubaker, M.A. (2017). cryoSPARC: algorithms for rapid unsupervised cryo-EM structure determination. Nat. Methods *14*, 290–296.

Rantalainen, K., Berndsen, Z.T., Murrell, S., Cao, L., Omorodion, O., Torres, J.L., Wu, M., Umotoy, J., Copps, J., Poignard, P., et al. (2018). Co-evolution of HIV envelope and apex-targeting neutralizing antibody lineage provides benchmarks for vaccine design. Cell Rep. *23*, 3249–3261.

Rathinakumar, R., Dutta, M., Zhu, P., Johnson, W.E., and Roux, K.H. (2012). Binding of anti-membrane-proximal gp41 monoclonal antibodies to CD4-liganded and -unliganded human immunodeficiency virus type 1 and simian immunodeficiency virus virions. J. Virol. *86*, 1820–1831.

Ringe, R.P., Ozorowski, G., Yasmeen, A., Cupo, A., Cruz Portillo, V.M., Pugach, P., Golabek, M., Rantalainen, K., Holden, L.G., Cottrell, C.A., et al. (2017). Improving the expression and purification of soluble, recombinant native-like HIV-1 envelope glycoprotein trimers by targeted sequence changes. J. Virol. *91*, e00264-17.

Ruprecht, C.R., Krarup, A., Reynell, L., Mann, A.M., Brandenberg, O.F., Berlinger, L., Abela, I.A., Regoes, R.R., Günthard, H.F., Rusert, P., and Trkola, A. (2011). MPER-specific antibodies induce gp120 shedding and irreversibly neutralize HIV-1. J. Exp. Med. *208*, 439–454.

Rutten, L., Lai, Y.T., Blokland, S., Truan, D., Bisschop, I.J.M., Strokappe, N.M., Koornneef, A., van Manen, D., Chuang, G.Y., Farney, S.K., et al. (2018). A universal approach to optimize the folding and stability of prefusion-closed HIV-1 envelope trimers. Cell Rep. *23*, 584–595.

Santos da Silva, E., Mulinge, M., and Perez Bercoff, D. (2013). The frantic play of the concealed HIV envelope cytoplasmic tail. Retrovirology *10*, 54.

Schoofs, T., Barnes, C.O., Suh-Toma, N., Golijanin, J., Schommers, P., Gruell, H., West, A.P., Jr., Bach, F., Lee, Y.E., Nogueira, L., et al. (2019). Broad and potent neutralizing antibodies recognize the silent face of the HIV envelope. Immunity *50*, 1513–1529.e1519.

Seabright, G.E., Doores, K.J., Burton, D.R., and Crispin, M. (2019). Protein and glycan mimicry in HIV vaccine design. J. Mol. Biol. *431*, 2223–2247.

Sliepen, K., Han, B.W., Bontjer, I., Mooij, P., Garces, F., Behrens, A.J., Rantalainen, K., Kumar, S., Sarkar, A., Brouwer, P.J.M., et al. (2019). Structure and immunogenicity of a stabilized HIV-1 envelope trimer based on a group-M consensus sequence. Nat. Commun. *10*, 2355.

Sok, D., and Burton, D.R. (2018). Recent progress in broadly neutralizing antibodies to HIV. Nat. Immunol. *19*, 1179–1188.

Sougrat, R., Bartesaghi, A., Lifson, J.D., Bennett, A.E., Bess, J.W., Zabransky, D.J., and Subramaniam, S. (2007). Electron tomography of the contact between T cells and SIV/HIV-1: implications for viral entry. PLoS Pathog. *3*, e63.

Steichen, J.M., Kulp, D.W., Tokatlian, T., Escolano, A., Dosenovic, P., Stanfield, R.L., McCoy, L.E., Ozorowski, G., Hu, X., Kalyuzhniy, O., et al. (2016). HIV vaccine design to target germline precursors of glycan-dependent broadly neutralizing antibodies. Immunity *45*, 483–496.

Stiegler, G., Kunert, R., Purtscher, M., Wolbank, S., Voglauer, R., Steindl, F., and Katinger, H. (2001). A potent cross-clade neutralizing human monoclonal antibody against a novel epitope on gp41 of human immunodeficiency virus type 1. AIDS Res. Hum. Retroviruses *17*, 1757–1765.

Sun, Z.Y., Oh, K.J., Kim, M., Yu, J., Brusic, V., Song, L., Qiao, Z., Wang, J.H., Wagner, G., and Reinherz, E.L. (2008). HIV-1 broadly neutralizing antibody extracts its epitope from a kinked gp41 ectodomain region on the viral membrane. Immunity *28*, 52–63.

Torrents de la Peña, A., and Sanders, R.W. (2018). Stabilizing HIV-1 envelope glycoprotein trimers to induce neutralizing antibodies. Retrovirology *15*, 63.

Torrents de la Peña, A., Julien, J.P., de Taeye, S.W., Garces, F., Guttman, M., Ozorowski, G., Pritchard, L.K., Behrens, A.J., Go, E.P., Burger, J.A., et al. (2017). Improving the immunogenicity of native-like HIV-1 envelope trimers by hyperstabilization. Cell Rep. *20*, 1805–1817.

Torrents de la Peña, A., Rantalainen, K., Cottrell, C.A., Allen, J.D., van Gils, M.J., Torres, J.L., Crispin, M., Sanders, R.W., and Ward, A.B. (2019). Similarities and differences between native HIV-1 envelope glycoprotein trimers and stabilized soluble trimer mimetics. PLoS Pathog. *15*, e1007920.

Tran, E.E., Borgnia, M.J., Kuybeda, O., Schauder, D.M., Bartesaghi, A., Frank, G.A., Sapiro, G., Milne, J.L., and Subramaniam, S. (2012). Structural mechanism of trimeric HIV-1 envelope glycoprotein activation. PLoS Pathog. *8*, e1002797.

Voss, N.R., Yoshioka, C.K., Radermacher, M., Potter, C.S., and Carragher, B. (2009). DoG Picker and TiltPicker: software tools to facilitate particle selection in single particle electron microscopy. J. Struct. Biol. *166*, 205–213.

Wang, Y., Kaur, P., Sun, Z.J., Elbahnasawy, M.A., Hayati, Z., Qiao, Z.S., Bui, N.N., Chile, C., Nasr, M.L., Wagner, G., et al. (2019). Topological analysis of the gp41 MPER on lipid bilayers relevant to the metastable HIV-1 envelope prefusion state. Proc. Natl. Acad. Sci. USA *116*, 22556–22566.

Ward, A.B., and Wilson, I.A. (2017). The HIV-1 envelope glycoprotein structure: nailing down a moving target. Immunol. Rev. *275*, 21–32.

Williams, L.D., Ofek, G., Schätzle, S., McDaniel, J.R., Lu, X., Nicely, N.I., Wu, L., Lougheed, C.S., Bradley, T., Louder, M.K., et al. (2017). Potent and broad HIV-neutralizing antibodies in memory B cells and plasma. Sci. Immunol. *2*, eaal2200.

Yuan, M., Cottrell, C.A., Ozorowski, G., van Gils, M.J., Kumar, S., Wu, N.C., Sarkar, A., Torres, J.L., de Val, N., Copps, J., et al. (2019). Conformational plasticity in the HIV-1 fusion peptide facilitates recognition by broadly neutralizing antibodies. Cell Host Microbe *25*, 873–883.e875.

Zhang, K. (2016). Gctf: real-time CTF determination and correction. J. Struct. Biol. *193*, 1–12.

Zhang, L., Irimia, A., He, L., Landais, E., Rantalainen, K., Leaman, D.P., Vollbrecht, T., Stano, A., Sands, D.I., Kim, A.S., et al.; IAVI Protocol G Investigators (2019). An MPER antibody neutralizes HIV-1 using germline features shared among donors. Nat. Commun. *10*, 5389.

Zheng, S.Q., Palovcak, E., Armache, J.P., Verba, K.A., Cheng, Y., and Agard, D.A. (2017). MotionCor2: anisotropic correction of beam-induced motion for improved cryo-electron microscopy. Nat. Methods *14*, 331–332.

Zivanov, J., Nakane, T., Forsberg, B.O., Kimanius, D., Hagen, W.J., Lindahl, E., and Scheres, S.H. (2018). New tools for automated high-resolution cryo-EM structure determination in RELION-3. eLife *7*, e42166.

Zwick, M.B., Labrijn, A.F., Wang, M., Spenlehauer, C., Saphire, E.O., Binley, J.M., Moore, J.P., Stiegler, G., Katinger, H., Burton, D.R., and Parren, P.W. (2001). Broadly neutralizing antibodies targeted to the membrane-proximal external region of human immunodeficiency virus type 1 glycoprotein gp41. J. Virol. *75*, 10892–10905.

STAR★METHODS

KEY RESOURCES TABLE

REAGENT or RESOURCE	SOURCE	IDENTIFIER
Antibodies		
Monoclonal anti-HIV-1 Env PGT151-TEV	(Lee, Ozorowski, and Ward 2016)	N/A
Monoclonal anti-HIV-1 Env PGT121	NIH AIDS Reagent Program; https://www.hiv.lanl.gov/	RRID: AB_2491041
Monoclonal anti-HIV-1 Env DH511.2	(Williams et al., 2017)	KY272651.1 (HC); KY272652.1 (LC)
Monoclonal anti-HIV-1 Env VRC46.01	(Krebs et al., 2019)	N/A
Monoclonal anti-HIV-1 Env VRC42.01	(Krebs et al., 2019)	N/A
Monoclonal anti-HIV-1 Env VRC42.N1	(Krebs et al., 2019)	N/A
Monoclonal anti-HIV-1 Env 10E8v4_5R+100cF	(Kwon et al., 2018b)	N/A
Monoclonal anti-HIV-1 Env PGZL1	(Zhang et al., 2019)	N/A
Monoclonal anti-HIV-1 Env 10E8	(Huang et al., 2012)	RRID: AB_2491067
Monoclonal anti-HIV-1 Env PGT145	NIH AIDS Reagent Program; https://www.hiv.lanl.gov/	Cat#12703; RRID: AB_2491054
Monoclonal anti-HIV-1 Env VRC01	NIH AIDS Reagent Program; https://www.hiv.lanl.gov/	Cat#12033; RRID: AB_2491019
Monoclonal anti-HIV-1 Env 39F	NIH AIDS Reagent Program; https://www.hiv.lanl.gov/	Cat#11437
Bacterial and Virus Strains		
E. Coli BL21(DE3)	Sigma-Aldrich	Cat#CMC0014
Chemicals, Peptides, and Recombinant Proteins		
Peptidisc scaffold	Peptidisc Biotech	N/A
Isopropyl β-D-1-thiogalactopyranoside (IPTG)	Sigma-Aldrich	Cat#I6758
Teknova HBS-P BUFFER PH 7.4	Fisher Scientific	Cat#NC0804628
n-Dodecyl β-D-maltoside (DDM)	Sigma-Aldrich	Cat# D4641
CHAPS detergent	Sigma-Aldrich	Cat#10810118001
Sodium deoxycholate detergent	Sigma-Aldrich	Cat#D6750
PEI Max transfection reagent	PolySciences, Inc	Cat# 24765-1
Triton X-100 detergent	Sigma-Aldrich	Cat#11332481001
Opti-MEM	Thermo Fisher	Cat#31985070
18:1 (Δ9-Cis) 1,2-dioleoyl-sn-glycero-3-phosphocholine (DOPC)	Avanti Polar Lipids	Cat#850375
18:1 1,2-dioleoyl-sn-glycero-3-phospho-L-serine (DOPS)	Avanti Polar Lipids	Cat#840035
18:1 (Δ9-Cis) 1,2-dioleoyl-sn-glycero-3-phosphoethanolamine (DOPE)	Avanti Polar Lipids	Cat#850725
18:1 PA 1,2-dioleoyl-sn-glycero-3-phosphate (DOPA)	Avanti Polar Lipids	Cat#840875
18:1 1,2-dioleoyl-sn-glycero-3-phospho-(1'-myo-inositol-4',5'-bisphosphate) (PIP2(4,5))	Avanti Polar Lipids	Cat#840046
18:1 (Δ9-Cis) 1,2-dioleoyl-sn-glycero-3-phospho-(1'-rac-glycerol) (DOPG)	Avanti Polar Lipids	Cat#840475
cholesteryl hemisuccinate (CHS)	Sigma-Aldrich	Cat#C6512
Amphipol A8-35	Anatrace	Cat#A835

(*Continued on next page*)

Continued

REAGENT or RESOURCE	SOURCE	IDENTIFIER
Deposited Data		
PC64FL + PGT151 Fab + VRC42.01 Fab – micelle – cryo-EM	The Electron Microscopy Data Bank	EMDB-21321
AMC011FL + PGT151 Fab + VRC42.01 Fab – micelle – cryo-EM	The Electron Microscopy Data Bank	EMDB-21322
PC64FL + PGT151 Fab + VRC42.N1 Fab – micelle – cryo-EM	The Electron Microscopy Data Bank	EMDB-21323
PC64FL + PGT151 Fab + DH511.2 Fab – micelle – cryo-EM	The Electron Microscopy Data Bank	EMDB- 21324
PC64FL + PGT151 Fab + VRC46.01 Fab – micelle – cryo-EM	The Electron Microscopy Data Bank	EMDB- 21326
AMC011FL + PGT151 Fab + PGZL1 Fab – micelle – cryo-EM	The Electron Microscopy Data Bank	EMDB- 21327
AMC011FL + PGT151 Fab + 10E8v4-5R 100cF Fab – micelle – cryo-EM	The Electron Microscopy Data Bank	EMDB- 21328
BG505delCT + PGT151 Fab – Nanodisc – Cryo-EM	The Electron Microscopy Data Bank	EMDB- 21329
BG505delCT (Ectodomain) + PGT151 Fab	The Electron Microscopy Data Bank	EMDB- 21330
PC64FL + PGT151 Fab – Nanodisc – Cryo-EM	The Electron Microscopy Data Bank	EMDB-21331
AMC011FL + 2 X PGT151 Fab + 1 X 10E8 Fab	The Electron Microscopy Data Bank	EMDB-21332
AMC011FL + 2 X PGT151 Fab + 3 X 10E8 Fab	The Electron Microscopy Data Bank	EMDB- 21333
AMC011FL + 1 X PGT151 Fab + 2 X 10E8 Fab	The Electron Microscopy Data Bank	EMDB- 21334
AMC011FL + 1 X PGT151 Fab + 3 X 10E8 Fab	The Electron Microscopy Data Bank	EMDB- 21335
PC64FL - Bicelle – Negative stain EM	The Electron Microscopy Data Bank	EMDB- 21336
PC64FL + VRC42.01 Fab - Bicelle – Negative stain EM	The Electron Microscopy Data Bank	EMDB- 21337
PC64FL + DH511.2 Fab - Bicelle – Negative stain EM	The Electron Microscopy Data Bank	EMDB- 21338
PC64FL + PGT151 Fab - Nanodisc – Negative stain EM	The Electron Microscopy Data Bank	EMDB- 21339
PC64FL + VRC42.N1 Fab - Bicelle – Negative stain EM	The Electron Microscopy Data Bank	EMDB- 21340
AMC011FL + VRC42.01 Fab - Nanodisc – Negative stain EM	The Electron Microscopy Data Bank	EMDB- 21341
AMC011FL + 10E8 Fab - Nanodisc – Negative stain EM	The Electron Microscopy Data Bank	EMDB- 21342
BG505-ST-710 – Peptidisc – Negative stain EM	The Electron Microscopy Data Bank	EMDB- 21343
BG505-ST-710 + 10E8 Fab - Peptidisc – Negative stain EM	The Electron Microscopy Data Bank	EMDB- 21344
Hybrid model of AMC011FL + 1 X PGT151 Fab + 3 X 10E8 Fab – Nanodisc – Cryo-EM	Protein Data Bank	PDB- 6VPX
Experimental Models: Cell Lines		
FreeStyle HEK293F	Thermo Fisher	Cat#R79007
Recombinant DNA		
MSP1D1 scaffold expression plasmid	http://www.addgene.org (Denisov et al., 2004)	Cat#20061

(*Continued on next page*)

Continued

REAGENT or RESOURCE	SOURCE	IDENTIFIER
PC64M18C043-FL (PC64FL) Expression plasmid	(Landais et al., 2017)	N/A
AMC011FL Expression plasmid	(Torrents de la Peña et al., 2019)	N/A
BG505ΔCT Expression plasmid	(Cao et al., 2018)	N/A
BG505-ST-710 DNA Expression plasmid	This paper	N/A
Software and Algorithms		
Leginon automated image collection software	Potter et al., 1999	RRID:SCR_016731
DogPicker	Voss et al., 2009	https://emg.nysbc.org/redmine/projects/software/wiki/DoGpicker
Relion 3.0	Zivanov et al., 2018	RRID:SCR_016274
cryoSPARC2	Punjani et al., 2017	RRID:SCR_016501
MotionCor2	Zheng et al., 2017	RRID:SCR_016499
GCTF	Zhang, 2016	SCR_016500
Multibody refinement	Nakane et al., 2018	RRID:SCR_016274
CHARMM	Jo et al., 2008	RRID:SCR_014892
USCF Chimera	Goddard et al., 2018	RRID:SCR_004097
USCF ChimeraX	Pettersen et al., 2004	RRID:SCR_015872
GraphPad Prism	N/A	RRID:SCR_002798
Other		
Superdex 200 increase 10/300 GL column	GE life sciences	Cat#28990944
0.22 μm PES bottle top filter	Thermo Fisher	Cat#596-3320
HiTrap protein A column	GE life sciences	Cat#17040301
HiTrap KappaSelect	GE life sciences	Cat#17545812
Biobeads SM-2 Resin	Bio-Rad	Cat#1523920
Methyl α-D-mannopyranoside	Sigma-Aldrich	Cat#M6882
Sepharose 4B resin	GE life sciences	Cat# 17012001
Ni-NTA matrix	QIAGEN	Cat# 30210
Protein A Sepharose Fast Flow	GE life sciences	Cat#17-0974-01
Lentil Lectin Sepharose 4B	GE life sciences	Cat#17044401
Streptavidin (SA) Biosensors	ForteBio	Cat#NC9658567
Biotinylated Galanthus Nivalis Lectin (GNL)	Vector Laboratories	Cat#B-1245
Carbon-coated Cu400 mesh grid	Electron Microscopy Sciences	Cat#EMS400-Cu
Quantifoil 1.2/1.3 Holey Carbon Grids	Electron Microscopy Sciences	Q410CR1.3
C-flat grids 1.2/1.3	Electron Microscopy Sciences	CF413
Graphene Oxide on Quantifoil Grids	Electron Microscopy Sciences	GOQ400R1213

RESOURCE AVAILABILITY

Lead Contact

Further information and requests for resources and reagents should be directed to and will be fulfilled by the Lead Contact, Andrew B. Ward (andrew@scripps.edu).

Materials Availability

Expression clone BG505-ST-710 is available from the Lead Contact without restriction.

Data and Code Availability

3D EM reconstructions have been deposited in the Electron Microscopy Databank (http://www.emdataresource.org/) or Protein Databank (http://www.rcsb.org) under the accession numbers listed in Table S1 and the Key Resources Table.

EXPERIMENTAL MODEL AND SUBJECT DETAILS

HEK293F embryonic kidney cells were cultured in Freestyle 293F expression medium as suspension cell cultures in a humidified incubator at +37°C, supplied with 8% CO^2. Cells were agitated at 135 rpm.

BL21(DE3) *E. coli* cells were cultured in Luria Broth (LB) medium at +37°C and agitated at 200 rpm.

METHOD DETAILS

Recombinant protein expression and purification

The MSP1D1 scaffold protein was expressed and purified according to standard *E. coli* recombinant protein expression methods using plasmid available at addgene (https://www.addgene.org, plasmid #20061) (Denisov et al., 2004). Briefly, BL21(DE3) *E. coli* cells were transfected with plasmid expressing the scaffold and grown to an OD_{600} of ~0.8. Expression was induced with 1mM IPTG for ~5 hours. Cells were harvested and MSP1D1 purified by Ni-NTA affinity purification followed by size exclusion using a Superose S6i 10/300 column. The MSP1D1 was used without His-tag cleavage throughout the study. Peptidisc scaffold was purchased from Peptidisc Biotech (https://peptidisc.com/).

Env clones PC64M18C043-FL (PC64FL), AMC011FL and BG505ΔCT were purified as described previously (Blattner et al., 2014; Rantalainen et al., 2018; Torrents de la Peña et al., 2019). Briefly, FreeStyle 293F cells (Thermo Fisher #R79007) cells were transfected with 250 μg of Env DNA per liter of cells and supplemented with 62.5 μg/ml of furin DNA to ensure complete cleavage of Env at cell density of 1.6 milj/ml. Cells were harvested 72 hours post transfection. PGT151 with an engineered TEV site between the Fab and Fc was added on cells prior to lysis with DDM containing buffer. Cleared lysate was mixed with protein A matrix and incubated overnight at +4°C. Next, matrix was washed with 50 mM Tris-HCl (pH 7.4), 300 mM NaCl, 0.1% (w/v) CHAPS, 0.03 mg/mL deoxycholate followed by wash with 50 mM Tris-HCl (pH 7.4), 500 mM NaCl, 0.1% (w/v) DDM, 0.03 mg/mL deoxycholate and finally exchanged to buffer with 50 mM Tris-HCl pH 7.4, 150 mM NaCl, 0.1% (w/v) DDM, 0.03 mg/mL deoxycholate and 2 mM EDTA in gravity flow column. Env was eluted by adding ~200 μg of TEV enzyme per liter of cell culture used and incubated for 4h at room temperature. Sample was then concentrated and purified with size exclusion chromatography using a Superose S6i 10/300 column.

BG505-ST-710 DNA construct, codon optimized for mammalian cell expression, was subcloned into a pcDNA3.4 expression vector. 293F cells were co-transfected with BG505-ST-710 and furin DNA vectors (500 and 250 μg per 1 L of cells, respectively) using PEI (PolySciences, Inc). The cells were harvested by centrifugation (3,000 RCF, 30 min, 4°C) 48 – 96 hours post-transfection and lysed using 25 mM Tris + 300 mM NaCl + 1% Triton X-100 buffer (pH 7.4) for 2 hours at 4°C. Cell lysates were cleared by centrifugation (12,000 RCF, 1 hour, 4°C) and subsequent vacuum-filtration (0.22 μm PES filter, Thermo Fisher). Cleared lysates were run over a Sepharose 4B resin (GE Life Sciences) with immobilized PGT145, 2G12 or PGT151 antibodies. BG505-ST-710 was eluted off the column using the elution buffer 25 mM Tris + 3 M MgCl2 + 0.05% DDM + 0.003% DOC (pH 7.2). Sample was concentrated, buffer-exchanged to the gel-filtration buffer 25 mM Tris + 300 mM NaCl + 0.05% DDM + 0.003% deoxycholate and subjected to SEC (Superose S6i 10/300 column).

IgG and Fab expression and purification

IgGs and Fabs were expressed following standard protocols as follows. Proteins were expressed in FreeStyle 293F cells (Thermo Fisher #R79007). About 25 mL Opti-MEM (Thermo Fisher #31985070) containing ~750 μg DNA (500 μg heavy chain and 250 μg light chain plasmid) for Fab or ~500 μg DNA (250 μg heavy chain and 250 μg light chain plasmid) for IgG was mixed with 25 mL Opti-MEM containing 2,250 μg polyethylene imine MAX (MW 40,000; Polyscience - 24765-1). After incubation for 20 min at RT, the transfection mix was added to 1L cells at a density of ~1.2x106 cells/ml in FreeStyle 293 Expression Medium (Thermo Fisher - 12338018). The cells were incubated at 37°C and 8% CO2 for 6 days. After harvesting the cells, the supernatant, containing IgG or Fab, was filtered and loaded into a HiTrap protein A column (GE Life Sciences #17040301, for IgG) or HiTrap KappaSelect column (GE Life Sciences #17545812, for Fab). The column was washed with phosphate buffered saline and eluted with 0.1 M glycine pH 2.7. The fractions were concentrated, and the buffer was changed to 20 mM sodium acetate pH 5.5. The Fab was loaded into a Mono S column and was eluted with a 0 to 60% linear gradient of 1M sodium chloride in 20 mM sodium acetate pH 5.5 buffer. The Fabs were concentrated and stored in 20 mM sodium acetate pH 5.5, PBS or TBS at 4°C or at −80°C.

Lipid stock preparation

To ensure reproducibility, all lipid stock solutions were prepared as follows: lipids were either first dissolved in chloroform or, when available, in solvent used from pre-dissolved ampules. Solvent was evaporated for ~20 minutes under nitrogen gas flow and slow rotation until lipid was deposited as a thin film on the surface of the ampule. Lipids were next re-solubilized into lipid rehydration buffer containing 50 mM Tris (pH 7.4), 150 mM NaCl and 0.1% DDM, and diluted to a final concentration of 1 mM. After 30 min incubation at room temperature, stock solutions were sonicated using stepped micro tip (3 mm), 20 - 25% efficiency and 50% time cycle until the solution was clear (5-30 mins). Stocks were then aliquoted and stored in −80C° until use for up to 6 months. The following lipids were used throughout the study: 18:1 (Δ9-*Cis*) 1,2-dioleoyl-sn-glycero-3-phosphocholine

CellPress
OPEN ACCESS

(DOPC), 18:1 1,2-dioleoyl-sn-glycero-3-phospho-L-serine (DOPS), 18:1 (Δ9-*Cis*) 1,2-dioleoyl-sn-glycero-3-phosphoethanolamine (DOPE), 18:1 PA 1,2-dioleoyl-sn-glycero-3-phosphate (DOPA), 18:1 1,2-dioleoyl-sn-glycero-3-phospho-(1'-myo-inositol-4',5'-bisphosphate) (PIP2(4,5)), 18:1 (Δ9-*Cis*) 1,2-dioleoyl-sn-glycero-3-phospho-(1'-rac-glycerol) (DOPG), and cholesteryl hemisuccinate (CHS). The lipid mix for disc assembly was prepared by mixing the thawed lipid stocks to final ratio (see assembly below for details), followed by 3 times freeze-thaw cycle with LN2 and room temperature and vortexing between freeze-thaw cycles to ensure even mixing of lipids in DDM micelles.

Assembly

An overview of protein purification and assembly workflow is presented in Figure 1A. Protein concentrations were calculated using absorbance at 280nm and corrected with protein specific extinction coefficient factors. Purified Env was concentrated to ~1mg/mL using Amicon concentrators with 100 kDa molecular weight cut off (MWCO) prior to mixing with other components. The lipid mixture stock solution was prepared to a total concentration of 1mM. Typical molar ratio for different lipids in the mixture was 40:40:20 (DOPC:DOPS:CHS). MSP1D1 and peptidisc scaffold were diluted to 2mg/mL stocks. Molar ratios of disc assembly components were screened yielding following standard conditions for the assembly mix: 1:8:240 (Env:scaffold:lipid) for MSP1D1 scaffolded discs and bicelles and 1:120:580 for peptidisc. A typical reaction for MSP1D1 scaffolded assemblies consisted of 25 μL of Env, 25 μL of lipids and 10 μL of MSP1D1 scaffold (tot 60 μL) or 25 μL of Env, 50 μL of lipid mix and 25 μL of peptidisc scaffold (100 μL) at concentrations given above. Assembly mixtures were incubated for ~30 min at room temperature prior to assembly initiation by addition of bio-beads. Prior to use, the bio-beads were rinsed with methanol for ~1 min followed by three consecutive washes with water (~10 X vol of the biobeads) and stored at +4C° for up to three days. Approximately 50% vol to total reaction volume of bio-beads was added per assembly reaction. The reaction was incubated for 24-48h at +4°C in a rotating mixer followed by transfer to a new tube with a new batch of bio-beads and additional incubation for 24h to maximize detergent removal. Purification of assembled discs was started by separating aggregated protein by centrifugation for 10 min at 13,000 x r*cf.* at +4°C on a tabletop centrifuge. Discs and bicelles were then subjected to a final polishing purification step either by lentil lectin Sepharose or size exclusion chromatography. Lentil lectin purification was initiated by mixing the assembled discs with TBS equilibrated, drained matrix (Lentil Lectin Sepharose 4B, GE life sciences) in a 1:1 sample-to-matrix ratio. The sample was allowed to bind overnight at +4C°, followed by 3 X washes with ~1.4ml TBS in a 1.5 mL test tube. Discs were eluted by three consecutive 1h incubations with TBS + 1 M methyl α-D-mannopyranoside using an equivalent volume of elution buffer to matrix (e.g., 100 μL elution buffer per 1h elution from 100 μL of drained lentil lectin matrix). Three fractions were pooled and dialyzed against Env-nanodisc buffer (20 mM Tris-HCl, pH 7.4, 40 mM NaCl) in a 20 kDa MWCO slide-a-lyzer dialysis units (Thermo Fisher Scientific) to remove methyl α-D-mannopyranoside and to prepare the sample for concentration by water evaporation (Savant DNA120 SpeedVac, Fisher Scientific). The sample was concentrated 5 to 10 times the original concentration depending on required protein concentration, resulting in the final sample for EM analysis. If size exclusion chromatography was used as the final polishing step, the Superose S6i 10/300 column was equilibrated in Env-nanodisc buffer and used according to the manufacturer's instructions. Unassembled Env, free scaffold, and lipids were separated, but peaks for different Env occupancies in discs were overlapped too much for separation and were pooled and concentrated as with the lectin purification method. The Env incorporation ratio was measured by calculating the amount of Env added to the assembly reaction versus the concentration in the purified final sample. The stability of disc preparations was assessed by measuring concentration of non-aggregated discs and with EM analysis either after storage in +4°C up to 6 months or by flash freezing in LN2 and storing at −80°C.

Mass spectrometric identification of lipids

Nanodisc solution (final 0.12 mg/mL, 40 μL) was digested with proteinase K (2:1, w/w) in TBS ammonium acetate buffer at 4°C for 2h or overnight, and diluted with final 40% (v/v) acetonitrile. Lipid LC MS/MS analyses were performed using an EASY-nLC connected with an LTQ-Orbitrap Velos mass spectrometer (Thermo Fisher). For each run, about 3 μL digest in 40% acetonitrile was directly loaded to a C8 analytical column (Phenomenex C8, bead diameter 5 μm, pore size 100Å, column inner diameter 100 μm, length 20 cm), and eluted with high %B gradients at 0.4 μL/min. LC buffer A was 0.1% formic acid/ 5% acetonitrile/H2O, and buffer B was 0.1% formic acid/ 95% acetonitrile/H2O.

MS instrument settings were adapted to those used for peptide analysis when applicable: spray voltage 2.5 kV and capillary temperature 325°C. CID MS/MS spectra were typically acquired using data-dependent or targeted manner in positive and negative ion modes for precursor m/z range 200-2000. Earlier experiments also acquired MS3 spectra to help confirm lipid identity. Data-dependent MS/MS used a top 10 method, in which one MS scan was followed by MS/MS scans of the top 10 most abundant MS peaks, and were measured by the linear ion trap analyzer using enhanced and normal scan speeds respectively. Ion trap scans had AGC on and used 1 micro scan and max ion injection time of 10 ms; MS/MS used isolation width 2 Th, normalized collision energy 35%, activation Q 0.20 and activation time 20 ms. Dynamic exclusion was applied at 1 Th width for 60 s duration and 2 repeats. The lipid-only vesicle sample formed from the input lipids without protein was used as a reference for lipid identification. Lipid identification was based on fragmentation analysis and comparison of MS and MS/MS spectra with those of the lipid-only vesicle sample and literature.

Bio-layer interferometry

Antigenic profiles of Env constructs were measured on an Octet Red 96 instrument (ForteBio) at 23°C in Octet buffer (1x HBS + 3 mM EDTA + 1 mg/mL BSA). Streptavidin (SA) Biosensors (ForteBio) were preincubated with 10 μg/mL biotinylated Galanthus Nivalis Lectin (Vector Laboratories) in Octet buffer for at least 10 minutes. BG505-based Env constructs were captured at 3 μg/mL in Octet buffer for 6 minutes. After a 30 s baseline phase, indicated Fabs were passed over the sensors for 180 s at 200 μg/mL, followed by a 180 s dissociation phase. Lectin sensors were regenerated using three 5 s incubations with 150 mM phosphoric acid. AMC001-based constructs showed lower signal than BG505, and were therefore measured in an inverted format in which IgGs were captured on Protein A chips and nanodiscs or micelles were flowed over the captured IgGs. Data from a matching negative control were subtracted from each curve, Y-axes were aligned to the baseline phase in the Octet Data Analysis software (version 11.0.2.3, ForteBio). Data were exported, normalized to the signal obtained for PGT121, and plotted in Prism for macOS 8.2.1 (Graphpad Software).

Electron microscopy sample preparation

Concentrations of final assemblies were estimated using absorbance measurement at 280 nm and extinction coefficient of full-length HIV Env, and verified by negative-stain EM thereby not including the absorbance coming from the scaffold protein. In samples where Env nanodiscs and bicelles were complexed with Fabs for negative-stain EM, three times molar excess of purified Fab fragment per Env trimer was incubated with the Env nanodiscs overnight at +4°C and stained as follows: 3 μL of purified disc preparation at 0.03 – 0.06 mg/mL was applied to a 400 mesh size Cu grid, blotted off with filter paper, and stained with 2% uranyl formate for 60 s. Cryo-EM grids were prepared differently depending on sample type: for lipid-detergent micelle 10 μL of Env at 5-7 mg/mL was mixed with 1 μL of 1 mM lipid mix and ~6 times molar excess of Fab, followed by gradual detergent removal and lipid replacement with three consecutive additions of 3-5 bio-beads with 1h incubation on ice between each addition. 3 μL of sample was then applied with 0.5 μL of 0.04% A8-35 amphiphol to either 1.2/1.3 Quantifoil or 1.2/1.3 C-flat grids and flash frozen in liquid ethane using Vitrobot mark IV (Thermo Scientific) without wait time, blot force of 0 and blot time of 6-7 s. PC64FL and BG505ΔCT nanodisc samples were frozen on 1.2/1.3 Quantifoil grids overlaid with in-house made thin carbon film at 0.1 – 0.5 mg/mL using Vitrobot without wait time, blot force of −10 and blot time of 2.5 – 3 s. AMC011FL nanodisc was prepared with lipid ratio 30:23:20:15:10:1:1 (DOPC:DOPS:CHS:DOPE: DOPA:PIP2(4,5):DOPG) and complexed with 10E8 Fab during the final lentil lectin polishing purification step of the nanodisc preparation. Approximately 10 X molar excess of Fab was added to matrix bound nanodiscs, incubated over night at +4°C, and washed extensively with TBS followed by elution, dialysis and concentrating steps as described above for nanodisc assembly. The sample was then frozen on graphene oxide grids (GO on Quantifoils R1.2/1.3, Cu, 400 mesh, Electron Microscopy Sciences) without wait time, blot force of 0, and blot time of 2.5 – 3 s at 0.1 – 0.2 mg/mL.

Electron microscopy imaging and data processing

Negative stain EM data was collected on a Tecnai Spirit microscope operating at 120 keV using Leginon automated image collection software (Potter et al., 1999). The image collection parameters are summarized in table S1. Cryo-EM data were collected on a Titan Krios and Talos Arctica operating at 300 keV or 200 keV, respectively, both equipped with a K2 direct electron detector (Gatan) using Leginon. Data were processed using various workflows depending on sample type as summarized in Figures S2– S4. In summary, particles from negative-stain EM data were picked using DogPicker (Voss et al., 2009) and exported to particle stacks using Relion 3.0 (Zivanov et al., 2018). 2D and 3D classification was done using cryoSPARC2 (Punjani et al., 2017). After one or two rounds of 2D classification and selection of particles with bicelle or disc-like features, approximately one reference free ab-initio 3D reconstruction was generated per 10,000 particles. Ab-initio models were then refined using homogeneous refinement option in cryoSPARC2. Cryo-EM data processing was initiated by frame alignment using MotionCor2 (Zheng et al., 2017), followed by contrast transfer function (CTF) calculation by GCTF (Zhang, 2016), particle picking with DogPicker, and extraction with Relion 3.0, or CTF calculation, particle extraction and 2D classification in cryoSPARC2. For detergent-lipid micelle datasets, particle stacks after 2D classification were exported to Relion 3.0 for subsequent processing steps. Here, initial particle coordinates were set by binning all imported particles by 4 and refining them using a 60 Å low-pass filtered Env ectodomain with two copies of PGT151 Fab as the initial model. This initial refinement was then used as a seed for subsequent one to four rounds of 3D classification before unbinning, final refinement, and postprocessing using Relion 3 standard parameters. Multibody refinement was done using the final, postprocessed 3D refinement as the starting model with Relion 3 multibody refinement function (Nakane et al., 2018). Nanodisc reconstructions were done in cryoSPARC2. Briefly, after one or two rounds of 2D classification, selected particles were classified using negative-stain reconstructions from the same sample as seed models with heterogeneous refinement function. This process was repeated using prior models as seeds when further classification was needed. Final, clean 3D classes were refined with non-uniform refinement and classes that refined beyond 7Å were postprocessed with local resolution estimation followed by local filtering. Angles between the membrane bilayer, Fab and Env were measures by placing first axis on the surface of the bilayer in EM density map, second axis through the center of the Fab density and fitted Fab structure, and the third through the 3-fold axis of the fitted Env structure. Height from bilayer in all data was estimated as follows. Env ectodomain structure model (e.g., PC64FL – PDB 6DCQ) was placed inside EM map using chimera fit in map -function. Next, a centroid was placed in the center of

last modeled residues (Asp664) of three Env protomers. Then, a bilayer modeled with CHARMM-GUI (Jo et al., 2008) was aligned to the EM density corresponding the nanodisc or bicelle. Distance from the centroid to the closest atom in the modeled bilayer was reported as the Env distance from bilayer. All EM data were visualized and analyzed using USCF Chimera and ChimeraX (Goddard et al., 2018; Pettersen et al., 2004). Hybrid model of AMC011FL nanodisc in complex with PGT151 Fab and 10E8 Fab was generated and deposited to Protein Data Bank with accession code PDB 6VPX. This model was generated by rigid body fitting separate gp120 and gp41 subunits and PGT151 variable domain of AMC011 FL structure (PDB: 6OLP), and variable domains of 10E8 Fab with MPER peptide (PDB: 5T80).

QUANTIFICATION AND STATISTICAL ANALYSIS

Signal normalization and graph presentation of bio-layer interferometry data was performed using GraphPad Prism software as described above.

Cell Reports, Volume *31*

Supplemental Information

HIV-1 Envelope and MPER Antibody Structures in Lipid Assemblies

Kimmo Rantalainen, Zachary T. Berndsen, Aleksandar Antanasijevic, Torben Schiffner, Xi Zhang, Wen-Hsin Lee, Jonathan L. Torres, Lei Zhang, Adriana Irimia, Jeffrey Copps, Kenneth H. Zhou, Young D. Kwon, William H. Law, Chaim A. Schramm, Raffaello Verardi, Shelly J. Krebs, Peter D. Kwong, Nicole A. Doria-Rose, Ian A. Wilson, Michael B. Zwick, John R. Yates III, William R. Schief, and Andrew B. Ward

Supplemental Information

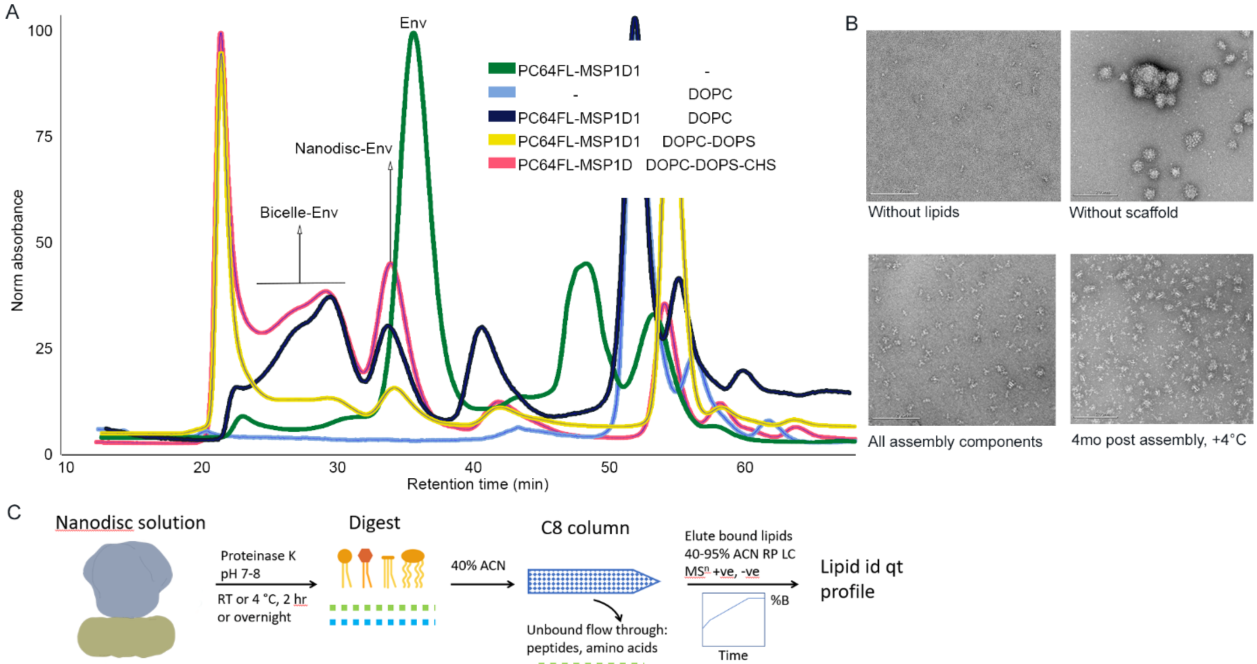

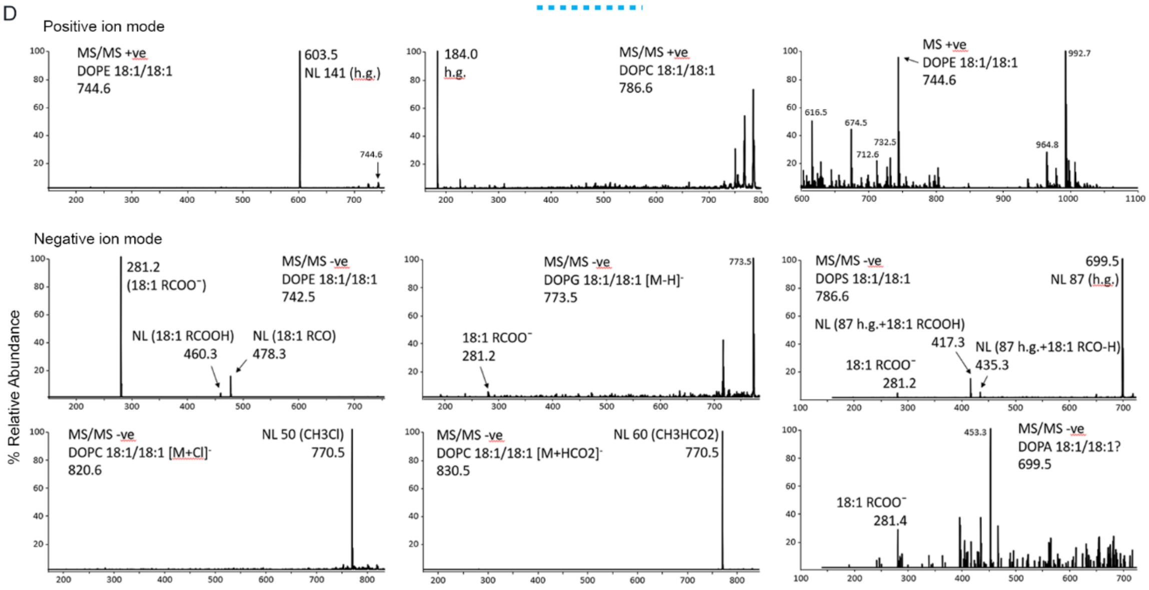

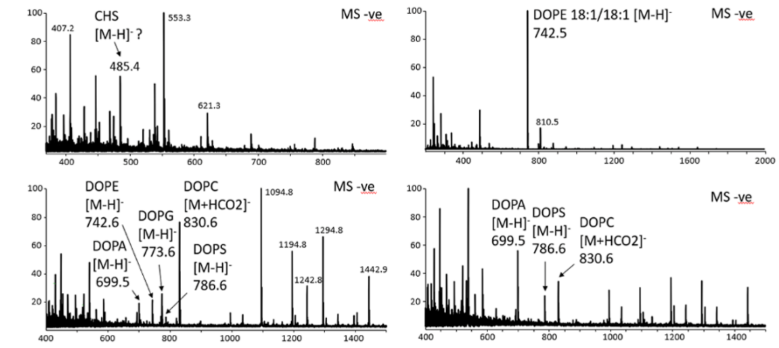

E

Identified lipids in nanodisc	Ion form	Exact mass m/z	MS/MS id signature	Ionization mode
**DOPE 18:1/18:1**	[M+H]+	**744.5538**	NL 141	positive
**DOPC 18:1/18:1**	[M+H]+	**786.6007**	184	positive
CHS	[M+Na]+	509.3607	not detected	positive (MS1)
DOPA 18:1/18:1	[M-H]-	699.4970	281	negative (MS1)
**DOPE 18:1/18:1**	[M-H]-	**742.5392**	281, NL 282, NL 264	negative
**DOPS 18:1/18:1**	[M-H]-	**786.5291**	NL 87	negative
**DOPG 18:1/18:1**	[M-H]-	**773.5338**	281	negative
**DOPC 18:1/18:1**	[M+Cl]-	**820.5624**	NL 50 (CH3Cl)	negative
	[M+HCO2]-	**830.5911**	NL 60 (CH3HCO2)	negative
PIP2 18:1	[M-H]-	1021.4825	not detected	negative
	[M-2H]2-	510.2376	not detected	negative
CHS	[M-H]-	485.3631	not detected	negative (MS1)

Figure S1 1. Controls for lipid assembly. Related to figure 1. A) Representative size exclusion chromatograms from a lipid mixture composition screen. B) representative raw micrographs of control reactions in the absence of scaffold or lipids and when all assembly components were present with an example of discs stored for 4 months at +4°C. C) Digest and conquer lipid analysis workflow. D) Selected disc lipid spectra in positive and negative ion mode. E) Disc lipid identification summary table. NL, neutral loss; h.g., head group; bold font, MS/MS confirmed.

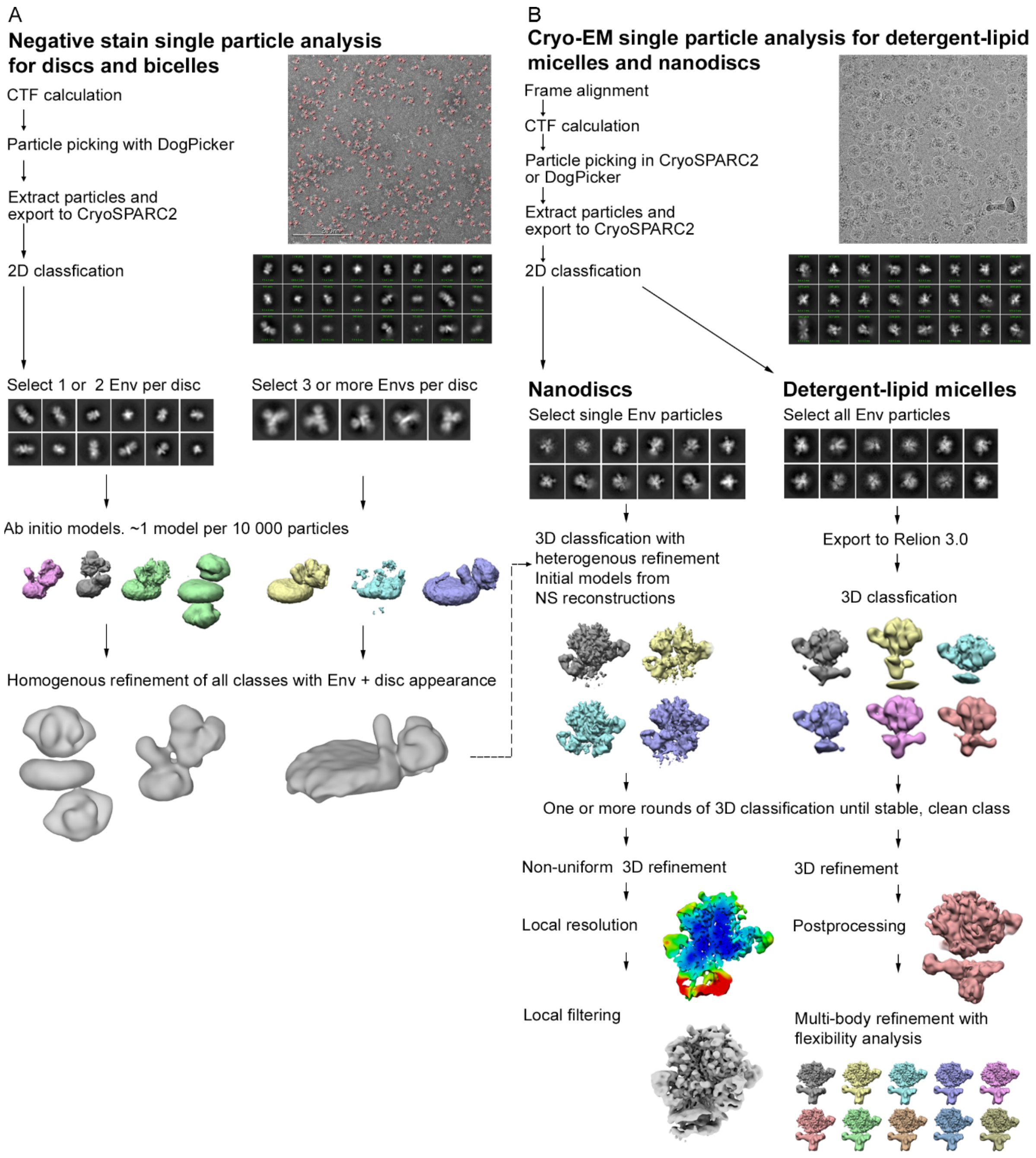

Figure S2. Single particle data processing workflows for analyzing different assemblies. Related to figures 1, 2, 3 and 4. A) negative stain EM, and B) cryo-EM.

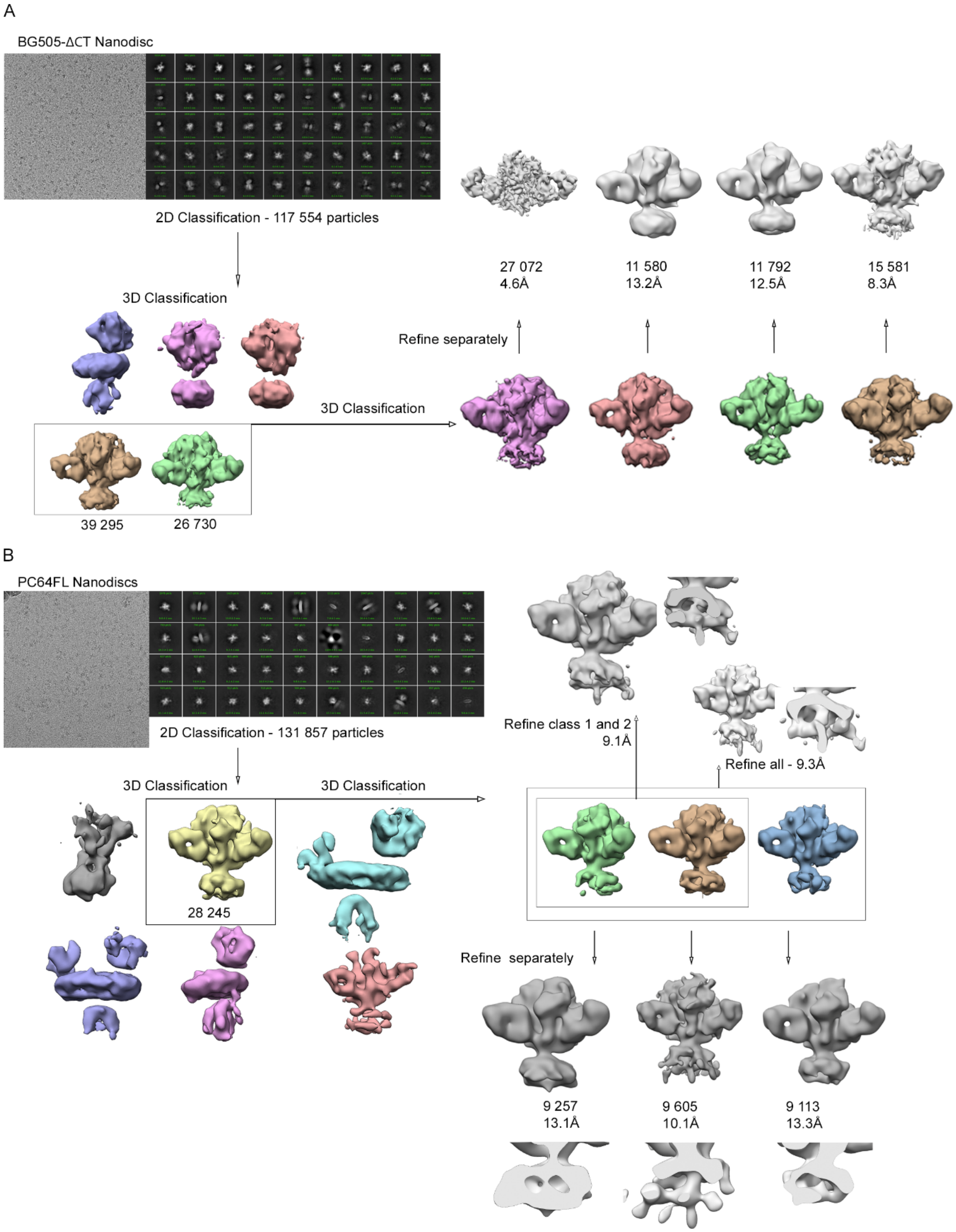

Figure S3. Representative raw micrographs and data processing workflows. Related to figure 2. A) BG505ΔCT and B) PC64FL nanodiscs. 3D classes with features of PGT151 stabilized ectodomain were selected for further 3D classification and refinement. In PC64FL embedded in nanodiscs, the additional membrane-embedded density was confirmed by refining the subclasses of nanodiscs independently.

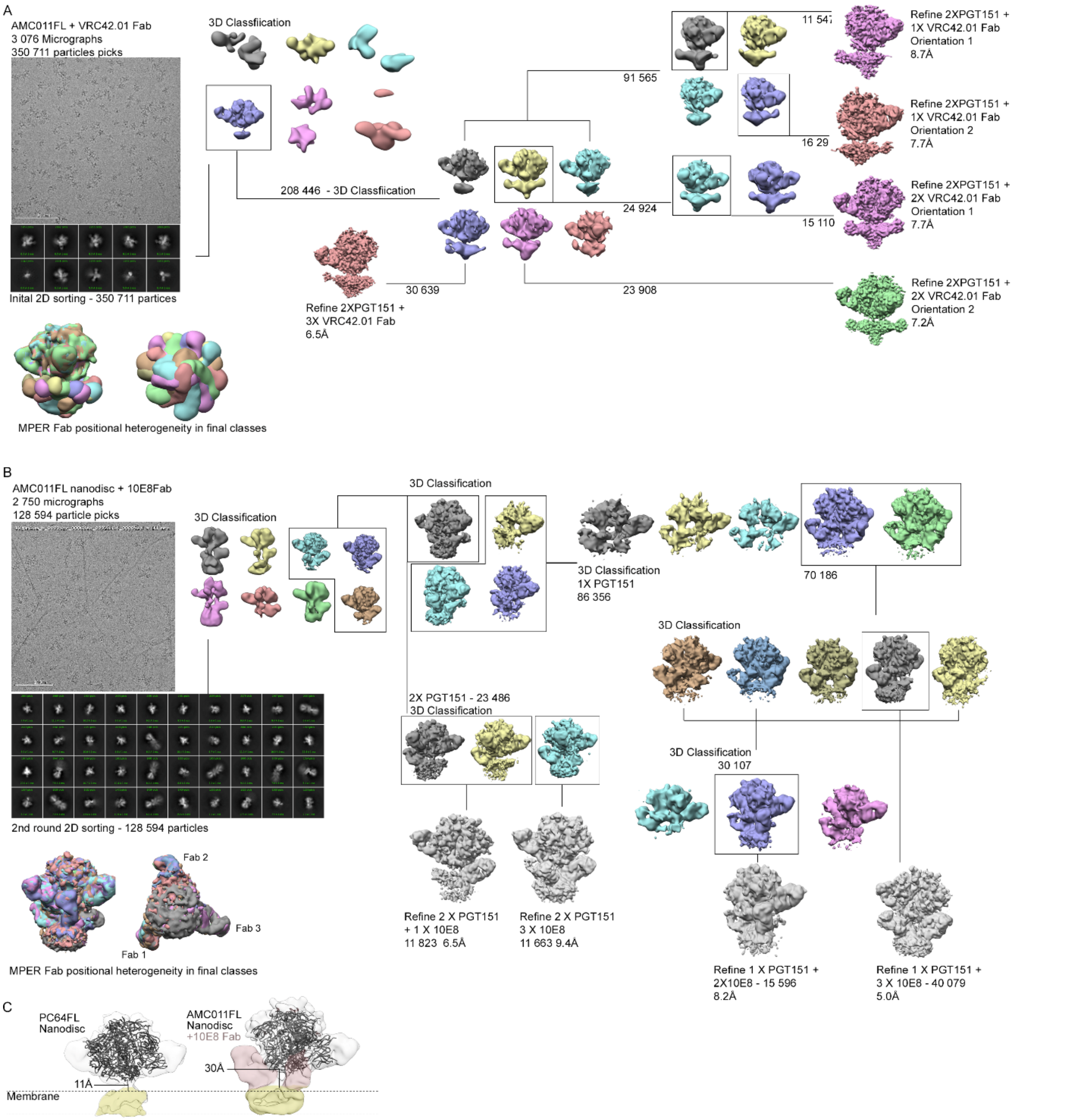

Figure S4. Comparison of data processing workflows. Related to figures 3 and 4. A) Detergent-lipid micelle approach and B) nanodisc approach. In the micelle approach, the MPER showed significant positional heterogeneity due to the flexible micelle and leading to near-continuous distribution of Fab positions. This, in combination with compositional heterogeneity originating from different Fab occupancy states led to significant reduction of particles in final, stable classes. In the nanodisc approach, the disc provides additional support for the Fab leading to three distinct and fixed MPER Fab positions thus reducing the heterogeneity and number of discarded particles. Classification for both approaches were tested in Relion 3.0 and CryoSPARC2. In the micelle samples, different Fab occupancy classes could not be efficiently separated by CryoSPARC2 heterogenous refinement whereas, with the nanodisc approach, both software provided comparable Fab occupancy separation. C) Comparison of ectodomain height in PC64FL nanodisc without MPER Fabs and AMC011FL nanodisc with 10E8 Fab. Height from the membrane surface is indicated.

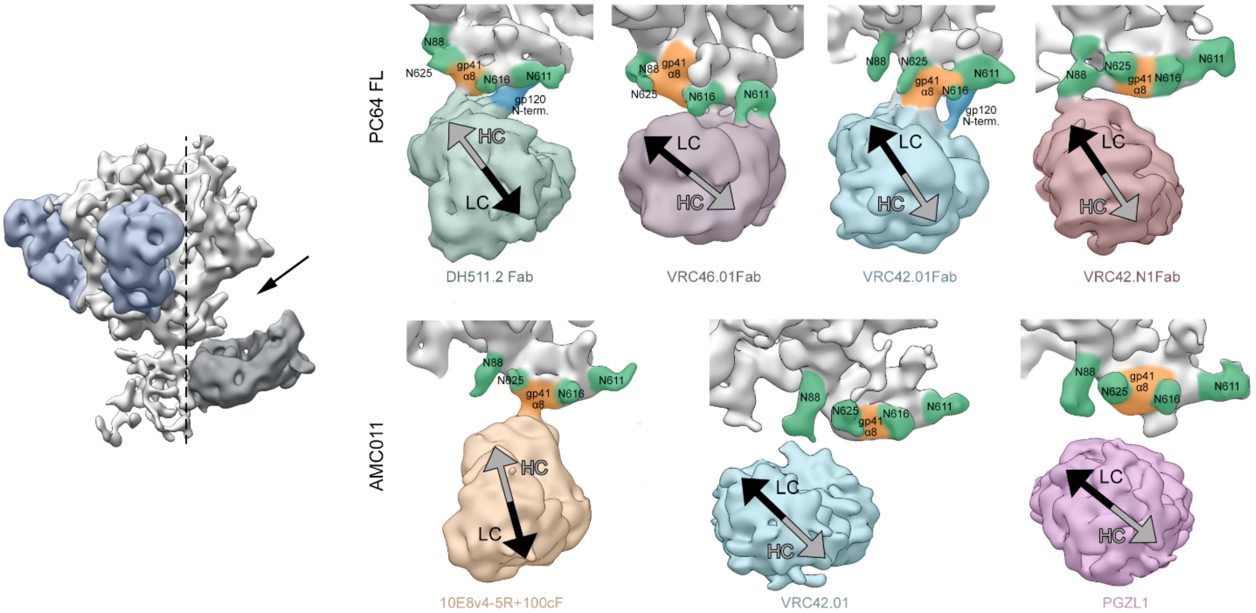

Figure S5. Proximity and positioning of MPER targeting Fabs in detergent-lipid micelle samples in relation to ectodomain. Related to figure 3. Densities corresponding to glycans, α8 helix and gp120 N-terminus are highlighted. Orientation of heavy (HC) and light chain (LC) are also indicated.

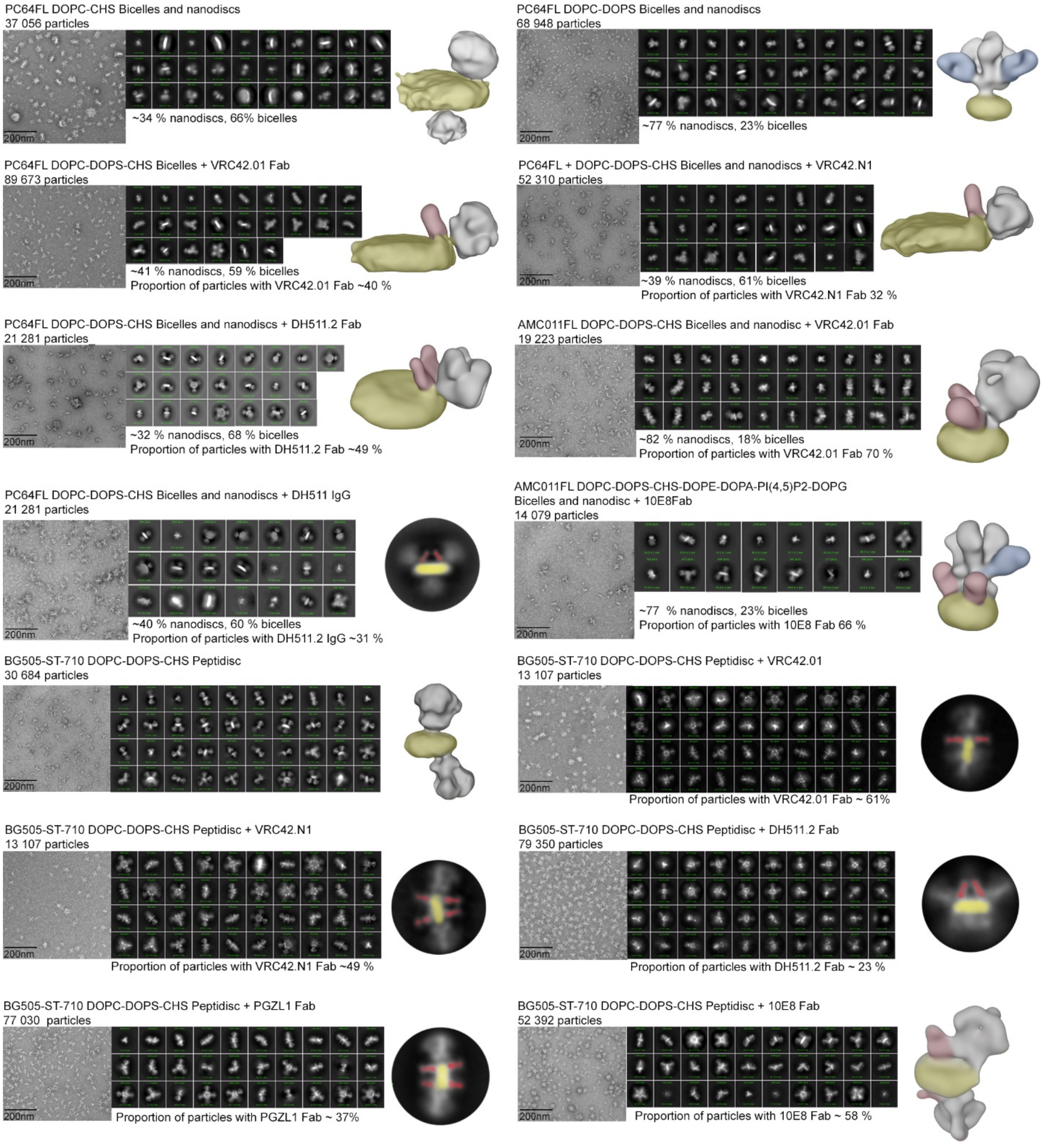

Figure S6. A panel of additional complexes analyzed as controls and as part of the method development. Related to figures 1, 2 and 4. Where data processing did not yield 3D reconstructions, matching features observed in 2D classes and representative 2D classes are shown. PGT151 Fab is highlighted in blue, MPER targeting Fab in red and the lipid bilayer in yellow. Proportion of nanodiscs and bicelles was estimated based on number of particles in 2D classes so that one and two Env particle classes with smaller diameter disc were counted as nanodiscs and the rest as bicelles. Proportion of MPER Fab bound particle was estimated based on particle number of combined 2D classes that have MPER Fab features for each dataset.

Table S1. Related to figures 3, 4 and 5. Summary of EM samples and imaging conditions.

**Sample Name**	Type of assembly	NS / Cryo	Microscope	Number of raw micrographs	Total number of Env particles	Number of particles used for reconstructions	Resolution (Å)	EMDB/PDB
PC64FL + PGT151 Fab + VRC42.01 Fab	Micelle	Cryo	Krios	8576	479 362	43 834	7.4	21321
AMC011FL + PGT151 Fab + VRC42.01 Fab	Micelle	Cryo	Krios	3078	350 711	23 908	7.2	21322
PC64FL + PGT151 Fab + VRC42.N1 Fab	Micelle	Cryo	Krios	2457	491 727	5 809	8.6	21323
PC64FL + PGT151 Fab + DH511.2 Fab	Micelle	Cryo	Krios	4463	748 662	7 661	7.5	21324
PC64FL + PGT151 Fab + VRC46.01 Fab	Micelle	Cryo	Krios	2288	340 716	30 378	9	21326
AMC011FL + PGT151 Fab + PGZL1 Fab	Micelle	Cryo	Krios	5788	279 853	21 264	6.6	21327
AMC011FL + PGT151 Fab + 10E8v4-5R 100cF Fab	Micelle	Cryo	Krios	6751	599 629	23 653	7.8	21328
BG505delCT + PGT151 Fab	Nanodisc	Cryo	Arctica	2343	117 554	13 324	9.9	21329
BG505delCT (Ectodomain) + PGT151 Fab	Nanodisc	Cryo	Arctica	2343	117 554	27 027	4.6	21330
PC64FL + PGT151 Fab	Nanodisc	Cryo	Arctica	3909	131 857	19 132	9.1	21331
AMC011FL + 2 X PGT151 Fab + 1 X 10E8 Fab	Nanodisc	Cryo	Arctica	2750	128 594	11 823	6.5	21332
AMC011FL + 2 X PGT151 Fab + 3 X 10E8 Fab	Nanodisc	Cryo	Arctica	2750	128 594	11 663	9.4	21333
AMC011FL + 1 X PGT151 Fab + 2 X 10E8 Fab	Nanodisc	Cryo	Arctica	2750	128 594	15 596	8.2	21334
AMC011FL + 1 X PGT151 Fab + 3 X 10E8 Fab	Nanodisc	Cryo	Arctica	2750	128 594	40 079	5	21335/6VPX
PC64FL	Bicelle	NS	Spirit	273	37 056	9 219	19	21336
PC64FL + VRC42.01 Fab	Bicelle	NS	Spirit	515	89 673	29 792	18	21337
PC64FL + DH511.2 Fab	Bicelle	NS	Spirit	440	21 281	11 154	20	21338
PC64FL + PGT151 Fab	Nanodisc	NS	Spirit	147	68 948	18 963	18	21339
PC64FL + VRC42.N1 Fab	Bicelle	NS	Spirit	417	52 310	23 785	17	21340
AMC011FL + VRC42.01 Fab	Nanodisc	NS	Spirit	106	19 223	5 004	19	21341
AMC011FL + 10E8 Fab	Nanodisc	NS	Spirit	240	14 079	4 172	26	21342
BG505-ST-710	Peptidisc	NS	Spirit	220	30 684	4 156	18	21343
BG505-ST-710 + 10E8 Fab	Peptidisc	NS	Spirit	403	52 392	17 567	20	21344
